# Leverage Points for Wellbeing and Achievement in Vocational Education: A Network Analysis of Psychological Factors Across Gender and Majors

**DOI:** 10.3390/bs16050706

**Published:** 2026-05-05

**Authors:** Maxim Likhanov, Adrien Fillon, Marie Demolliens, Anaïs Robert, Céline Darnon, Pascal Huguet, ProFAN Consortium, Isabelle Régner

**Affiliations:** 1 Centre de Recherche en Psychologie et Neurosciences (CRPN), Aix Marseille University, CNRS, 13003 Marseille, France; isabelle.regner@univ-amu.fr; 2 Université Clermont Auvergne, CNRS, LAPSCO, 63000 Clermont-Ferrand, France; adrien.fillon@uca.fr (A.F.); marie.demolliens@uca.fr (M.D.); anais.robert@uca.fr (A.R.); celine.darnon@uca.fr (C.D.); pascal.huguet@uca.fr (P.H.)

**Keywords:** gender differences, educational majors, vocational education, anxiety, psychometric networks, parental and school variables

## Abstract

The current study aimed to investigate complex links among a large set of anxiety-related variables and identify targets for well-being interventions in a large sample of male and female vocational education training students. In total, 28 psychological constructs, such as self-esteem, parental pressure and dissatisfaction and motivation, were assessed in four groups of VET students (mode age: 16). The sample included 3069 females in ASSP schools (nursing and caring); 2108 females and 1772 males in Commerce schools (sales and management); and 2262 males in MELEC schools (electricity and maintenance). We used Gaussian Graphical models (GGMs) that allow for building sparse models of links among multiple variables and detecting targets for interventions via the identification of the most central nodes. We showed gender differences in absolute means for some variables (higher self-esteem and math grades in males; higher anxiety and error sensitivity, but stronger endorsement of mastery approach achievement goals in females), as well as in network structure. GGMs suggested that the key nodes were self-reported math competence for females in the ASSP group, self-regulation for females in Commerce, and mastery approach goals for males in both MELEC and Commerce groups, and that these should be differentially targeted by educational interventions in these populations.

## 1. Introduction

Vocational education training (VET) is a large part of the educational system in France: 27.9% of high school students (627,100 out of 2,251,000) in 2023 enrolled in vocational education, compared to those in general and technological tracks (*Key Figures of the Education System*, 2023). Despite its importance for preparation of specialists that are ready to fill the demands of modern industries (Barnes et al., 2025; Bol & Van De Werfhorst, 2013; Van De Werfhorst & Mijs, 2010), VET is still perceived as a second—more negative—option in comparison with general education, including in France (European Centre for the Development of Vocational Training, 2017; Jonsson & Beach, 2015; Weller et al., 2025). This negative image could lead to a number of emotional problems in students in these schools, which are indeed demonstrated in previous studies that reported higher anxiety and lower well-being and self-esteem for VET students (see, e.g., a scoping review; Solberg et al., 2023). Moreover, 60% of students enrolled in VET have not chosen the vocational stream to which they are assigned as their first choice. This lack of choice may be a cause of school dropout and deviant behavior (Arrighi & Gasquet, 2010; Bernard & Troger, 2012; Jellab, 2017). Although clearly important, research in this area remains scarce, particularly in France, where only a few studies are available; however, these studies highlight the need to support VET students due to their lower school engagement and well-being (Arrighi & Gasquet, 2010; Bernard & Troger, 2012; Girard et al., 2022; Jellab, 2017).

Research into the well-being of VET students requires a multifactorial approach, given the complexity of the links among socio-emotional competences of students, school adjustment, anxiety, and achievement (e.g., Mella et al., 2021; Papageorgiou et al., 2020; Svraka et al., 2024). Importantly, such research should also consider gender and educational tracks, as previous studies have documented differences in anxiety-related constructs as a function of these variables (M. Likhanov et al., 2024a; M. V. Likhanov et al., 2021; Papageorgiou et al., 2020).

The current study aimed to investigate the structure of the interconnections among various anxiety-related concepts in male and female students from different educational majors, and to identify targets for educational interventions by means of network analysis.

### 1.1. School Anxiety and Its Psychological Consequences

School anxiety is defined as a response pattern, which includes unpleasant thoughts and apprehension, high level of arousal, and avoidance and/or escape behavior, that is elicited by stressful school environment (e.g., speaking to the class, being bullied or rejected by peers, and answering teachers’ questions) that the student perceives as threatening and/or dangerous (Delgado et al., 2019; García-Fernández et al., 2008). For example, an OECD’s Program for International Student Assessment (PISA) 2015 showed that 47.2% of French students reported feeling anxious even when they are well prepared for a test, and 29.2% get very tense when they study (OECD, 2017). Importantly, school anxiety is negatively linked with academic achievement (OECD, 2017), with high-anxiety students tending to earn lower grades and test scores (M. Likhanov et al., 2026) and this link being more pronounced in mathematics (Namkung et al., 2019). Academic-related anxiety is thus recognized as a risk factor for underachievement, making its reduction a key priority for improving student outcomes and well-being (Coates McDowall et al., 2025; Sammallahti et al., 2023).

School anxiety is linked not only to academic achievement but also to a number of motivational and self-belief variables, including lower academic motivation (Wang et al., 2015) and diminished self-esteem (Nguyen et al., 2019), as well as a poorer academic self-concept (Morales et al., 2023). Students’ mindsets and goals might also be associated with anxiety: those who endorse a fixed implicit theory of intelligence (believing ability is immutable) often experience greater anxiety about schoolwork, whereas a growth mindset is associated with more adaptive coping and higher academic performance and less anxiety (OECD, 2023; Ruiselová & Prokopčáková, 2005). Further, adolescents adopting performance-avoidance motivations (aiming to avoid looking incompetent) show significantly higher test anxiety than those with mastery-oriented goals (Möcklinghoff et al., 2023). Performance-avoidance goals have been consistently associated with negative academic outcomes, including lower achievement and increased anxiety or fear of failure (Bakadorova & Raufelder, 2020; Elliot & McGregor, 1999; Fiévé et al., 2025; Wirthwein et al., 2013). Youths high in error sensitivity—a perfectionistic tendency to view mistakes as intolerable threats—are more prone to anxiety (Hamilton & Kidwell, 2025). In addition, there is evidence linking school anxiety to impostor syndrome feelings: high-anxious high achievers frequently report believing they are frauds who will be “found out” (Fraenza, 2016).

Social-contextual factors contribute as well. Perceived parental pressure for high achievement is correlated with elevated student anxiety (Kulakow et al., 2021). Parental beliefs about a child’s math abilities are also related to the child’s math self-efficacy beliefs and performance level (X. Liu et al., 2025) and conceptions of intelligence (Gunderson et al., 2018). On the contrary, parental home support contributed positively to children’s performance on word problems and algebraic reasoning by reducing children’s mathematics anxiety (Vukovic et al., 2013). Previous research also suggested that teaching style and class environment may have an effect on achievement and interact with anxiety: the effect is weaker for some subjects (e.g., reading, English, and Chinese) than for mathematics (Carman & Zhang, 2012; Nye et al., 2004)—a high-stakes subject that is also associated with greater levels of student anxiety (Field et al., 2019). There was also an effect of the teacher on math anxiety, suggesting possible effects from teacher expectations, the teacher’s own anxiety, strictness, and other factors (Artemenko et al., 2021; Beilock et al., 2010). Further, social competence appears inversely related to school anxiety, with children who have stronger social skills and peer relations reporting significantly less anxiety in school settings (M. V. Likhanov et al., 2021; Magelinskaitė et al., 2014).

In sum, school anxiety is entwined with a broad network of interacting individual dispositions and environmental influences. Each of these factors could be a leverage point for interventions to reduce anxiety and improve students’ overall school adjustment and well-being.

### 1.2. Psychometric Networks as a Multifactorial Approach

A useful approach to investigate links among multiple interacting psychological constructs within one sparse model is a network analysis (Borsboom et al., 2021). This analysis allows for assessing associations (network edges) in large sets of variables (nodes; see discussion in Briganti et al., 2024) and has been applied in different domains, including personality (Costantini et al., 2015; Cramer et al., 2012; Le Tirant et al., 2026; Ruth et al., 2023) and cognitive ability (Conte et al., 2020; M. Likhanov et al., 2022, 2024b; Van Der Maas et al., 2006). For example, one study using network analysis showed that difficulties in school adaptation (poor adjustment to the academic and social environment) are linked to higher anxiety symptoms (Shalayiding et al., 2024).

Network perspective also allows looking into potential candidates for interventions, with high centrality nodes being considered as more promising candidates for manipulation/intervention than others in the treatment of mental disorders (Cramer & Borsboom, 2015). For example, one study identified sleep dissatisfaction, poor sleep quality, and uncontrollable worry as core symptoms (high on Expected Influence centrality metric) in the interplay among depression, anxiety, and sleep disturbance using network analysis (Tao et al., 2024); another one showed that affecting sleep quality is more effective to treat insomnia and depression compared to targeting depression symptoms in a randomized trial (Blom et al., 2015). This approach will be used in the current study to investigate connections and identify targets for interventions among a large number of anxiety-related variables in order to improve the overall well-being of students.

### 1.3. Educational Track and Gender Differences

Previous research has indicated that there are some educational track/major differences (e.g., STEM vs. literature students vs. their unselected peers) in absolute levels of anxiety and related constructs, such as personality, academic achievement, and self-reported creativity (M. Likhanov et al., 2024a; M. V. Likhanov et al., 2021; Papageorgiou et al., 2020; Repeykova et al., 2023; E. S. Tsigeman et al., 2023). For example, one recent study (Papageorgiou et al., 2020) investigated the structure of a network that featured personality, behavioral and emotional problems, and achievement in four samples of selected-for-achievement adolescents (STEM, Literature, Sports and Arts). The study showed that internalizing and externalizing problems were closely linked in the Sports sample, suggesting that young athletes tend to experience either both types of behavioral difficulties or neither, whereas other gifted individuals are more likely to experience more specific types of difficulties. The differences among educational majors are usually attributed to peculiarities of their environment (“high-stakes” competitions or high expectations from parents/teachers), development/training of specific characteristics during education (e.g., maths ability in the STEM track) or selection procedures (e.g., only students with high verbal ability being selected for the Literature track or more resilient students being selected for the Sports traits).

Vocational specializations likely expose students to domain-specific stressors that go beyond the aforementioned general stigma associated with VET pathways (see some discussion in Salmela-Aro et al., 2008). For example, nursing tracks involve high emotional demands, including exposure to suffering and the need for emotion regulation, which are known risk factors for stress and burnout (Buschle & Gruber, 2018; Maslach & Leiter, 2016; Rudman & Gustavsson, 2012), and potentially result in heightened anxiety and Imposter syndrome symptoms. One example could be a recent study that showed impulsivity and depression to be key factors in academic procrastination networks in the medical student population (Huang et al., 2025). In contrast, fields such as electrical engineering or maintenance require high levels of technical precision and error avoidance, where even minor mistakes can have serious consequences, contributing to performance pressure and potentially elevated error sensitivity (Le Coze, 2022; Reason, 1990). Commerce-related tracks, meanwhile, often involve interpersonal and evaluative stressors, such as customer interactions and performance-based assessments, which have been linked to stress in service-oriented roles (Grandey et al., 2004) and could require high resilience and ability to self-regulate. Together, these domain-specific demands may interact with broader educational stigma to shape students’ well-being and engagement.

In addition, research has consistently shown that girls report higher levels of anxiety compared to boys (Else-Quest et al., 2006; Esbjørn et al., 2013; Ferguson et al., 2015; Gibeau et al., 2023; Madjar et al., 2018; Zahn-Waxler et al., 2008). Research has documented gender differences in related constructs, such as academic achievement, self-esteem, motivation, etc. (see e.g., M. Likhanov et al., 2024a; Repeykova et al., 2023). Network analysis has also proved to be useful in examining gender differences in symptomatology of complex developmental trauma and symptoms of posttraumatic stress (Birkeland et al., 2017; Smith et al., 2023), as well as in individual differences research (Papageorgiou et al., 2020), showing both similarities and differences in networks for males and females.

### 1.4. Current Study

The current study will utilize network analysis on data coming from the ProFAN study—a large-scale (more than 10,000 students took part) nationwide multi-lab longitudinal experiment launched by the French Ministry of Education (Rudmann et al., 2024). The study included participants majoring in three areas: Métiers de l’Électricité et de ses Environnements Connectés (henceforth MELEC)—specialists in electricity and maintenance, with 95% of all students being males; Accompagnement, Soins et Services à la Personne (ASSP)—specialists in care and nurturing, with 90% females; and Commerce—specialists in sales and accounting, where gender distribution is almost equal.

One study that utilized network analysis in the ProFAN dataset was based on a sub-sample of 3400 vocational high school students and investigated relationships among 11 measures, including socio-emotional competencies, school adjustment measures and grades in mathematics and French language (Mella et al., 2021). The study showed that self-regulation at school weighted the most strongly on the whole network and was the most important mediatory pathway for other nodes in the network. The current study extends this work by relying on a much larger dataset (~9000 students), covering distinct vocational tracks and by including 28 psychological constructs.

Thus, the aim of the current study was two-fold: (1) to investigate the structure of the interconnections among various anxiety-related concepts in male and female VET students with different majors, and (2) to identify targets for interventions to improve the overall well-being of students.

## 2. Materials and Methods

### 2.1. Participants

Ten thousand three hundred ninety-five (10,395) participants were recruited in 109 vocational schools in France that were selected by the French Ministry of Education and were willing to participate in a large-scale longitudinal ProFAN project (Riant et al., 2024; Rudmann et al., 2024). This analysis focuses on data from the initial baseline of Cohort 1 (which was collected in September–October 2017), and Cohort 2 (September–October 2018)—before the intervention was introduced to students.

Schools were divided into MELEC (electricity, industrial systems and maintenance), ASSP (nursing and care) and Commerce (sales, promoting and managing retail space) educational tracks. Given that we were primarily interested in gender and type of educational track differences, we excluded participants for whom this information was missing, which comprised a sample of 9439 participants. We decided not to investigate females for the MELEC school and males for ASSP schools, as the sample sizes for these two schools were very small (N = 39 and N = 170, respectively) and precluded usage of planned ANOVA and network analysis. This resulted in a final sample of 9211 participants, with Ns for each group being the following: females in the ASSP school (F_ASSP henceforth) were 3069; for females in the Commerce school (F_COMMERCE)—2108; for males in Commerce (M_COMMERCE)—1772; and for males in MELEC schools (M_MELEC)—2262. Such gender distribution is similar to one reported by the French Ministry for Education (DEPP—Ministère de l’éducation nationale, 2025; Jean-Luc Tavernier, 2022). The age of participants ranged from “less than 15 years” to “more than 20”, with the majority of participants being 16 years old (see Table S1 in Supplementary Materials for exact frequencies).

### 2.2. Procedure

The data were collected during school hours in the schools’ computer rooms. Students completed an online survey containing multiple scales, with a subset of them included in the current study (see below). The survey was presented online using a specially designed internet platform.

Ethical review and approval were not required for this study involving human participants, in accordance with applicable local legislation and institutional regulations, as the research was designed and conducted under the authority of the French Ministry of Education. In line with national and institutional requirements, written informed consent from participants’ legal guardians or next of kin was not required. This study was approved by each participating school and was implemented as part of the regular curriculum; consequently, student participation was mandatory.

### 2.3. Measures

Table 1 presents information on 28 constructs that were used in the current study. Total scores were computed by averaging the items associated with each construct across all measures; these total scores were used in subsequent analysis. All measures were adapted to French, validated and demonstrated sufficient reliability in previous research and in the current study. The only exceptions were External motivation for math and French and Rigid facet of Theory of intelligence, for which the reliability was low. However, we decided to retain these three scales to counterbalance intrinsic motivation and Theory of intelligence—malleable scales which demonstrated high reliability.

### 2.4. Data Preprocessing

Little’s MCAR test (conducted with *naniar* package; (Tierney & Cook, 2023)) showed that the data is not Missing Completely At Random (*p* < 0.05). After visual inspection of the UpSet plot obtained with the same package (Figure S1C) and study protocols, we learned that some data were missing because some participants were absent from school when the questionnaire was administered. We decided not to impute missing data, even though the percentage of missing data for some variables was around 12 percent (e.g., self-regulation; see Figure S1 in Supplementary Materials). Rather, we opted for listwise deletion of incomplete cases for network analysis. Even without imputation, each variable still included more than 1000 datapoints—which is more than sufficient (usually N > 250) according to empirical research on network analysis, especially one that uses EBIC-lasso regularization (Constantin et al., 2023; Epskamp et al., 2018; Jones et al., 2018). As network analysis requires multivariate normality of data (H. Liu et al., 2009), we decided to delete univariate outliers, as their presence complicates the achievement of multivariate normality (Leys et al., 2019). Outliers were deleted using the interquartile range (IQR), i.e., [25th percentile] − 1.5 × IQR and [75th percentile] + 1.5 × IQR (McGill et al., 1978). The number of outliers was quite significant for some variables (up to 120 participants for Math grade in F_ASSP; see Figure S2 in Supplementary Materials), and the data for males and females separately violated normality for some variables after the outliers were deleted (See Figure S3 in Supplementary Materials).

We used Welch-ANOVA (Field et al., 2012), which is robust to normality assumption violations in large (N > 1000) samples and is recommended to be used when there are unequal groups and/or violations of the homoscedasticity assumption (Blanca et al., 2017; Schmider et al., 2010). As ANOVA does not require cases to be complete, sample sizes for ANOVAs were generally larger than those for network analysis and varied as a function of data availability for each variable.

### 2.5. Statistical Approach for Network Analysis

Gaussian Graphical Model Networks (GGMs) were estimated using a lasso regularization via the graphical lasso algorithm (Epskamp et al., 2018; Epskamp & Fried, 2018). In GGMs, if two nodes (variables) are not connected, i.e., not linked by an edge (connection), this means that they are linearly independent (not correlated)—controlling for the other variables in the network (Lauritzen, 1996). We computed 4 separate networks: for females in ASSP, for males in MELEC, and for males and females in COMMERCE. The number of observations for different measures ranged in different samples, so after selecting complete cases (i.e., data present for all 28 variables), the overall sample reduced to 6677 participants, with N = 2385 for F_ASSP; N = 1587 for M_MELEC; N = 1583 for F_COMMERCE; and N = 1122 for M_COMMERCE. Multivariate normality was violated for all 4 groups; thus, we applied a nonparanormal transformation to network analysis to relax the normality assumption (Isvoranu et al., 2017; H. Liu et al., 2009).

In network psychometrics of personality, centrality indices quantify the relative importance of traits within a network. In the current study, we focused on Strength centrality that reflects the sum of the absolute edge weights connected to a node, identifying traits that are strongly and directly connected to many others—key for recognizing hubs with broad influence (Bringmann et al., 2019). In addition, we used the Expected Influence (1 step) metric that is an extension of strength but also accounts for negative links, with nodes high on EI having more positive links and those low on EI having more negative links. This metric was shown to successfully identify nodes that play important roles in the disorder’s course, whereas a naive strength metric might misidentify a symptom with many inhibitory links as central (see, e.g., Robinaugh et al., 2016), and is usually more stable compared to other indices (Castro et al., 2024; Hevey, 2018). These indices have been used to detect highly influential traits, as potential targets for intervention, under the assumption that altering central traits may produce widespread, system-level effects (Costantini et al., 2015; Bringmann et al., 2019).

Some recent research has argued the need to look into clusters (communities of nodes) and bridge symptoms that link these clusters (Bringmann et al., 2019), as psychological networks are usually closely connected and well-being interventions are mostly “fat-handed”—affect many traits at once (Eronen, 2020). In addition, despite network analysis allowing for the examination of complex systems involving many variables, the interpretability of network structures tends to decrease as the number of nodes increases (e.g., beyond 10–20 variables), making patterns harder to meaningfully discern (Epskamp et al., 2018). The definition of clusters allows us to get a broader picture. In the computed networks, we defined clusters using the Walktrap algorithm (Pons & Latapy, 2005). This method uses short random walks on the network, under the assumption that walks are more likely to remain within densely connected regions. Nodes that are frequently co-visited during these walks are grouped into the same cluster, resulting in communities characterized by stronger internal than external connections. Edge weights were taken into account in the clustering procedure. We then inferred Bridge Expected Influence, which sums the edge weights of a node linking to all nodes in other clusters (Jones et al., 2021) and allows for identifying bridge nodes, which facilitate connections between distinct network clusters and may serve as key intervention targets (Kaiser et al., 2021).

Node predictability quantifies the proportion of variance of each node that is explained within the network model. Nodes with zero predictability value cannot be predicted by the model, whereas nodes with a predictability value of 1 can be perfectly predicted (Haslbeck & Waldorp, 2018). Theory of rigid intelligence was the node with the lowest predictability across all groups (R^2^ ranged from 0.09 to 0.13)—probably a result of the somewhat low reliability of the measure. Node predictability is visualized as rings (pie charts) around nodes in Figure 2 (Section 3.2.1). The nodes with the highest predictability are listed in Table 2 (Section 3.2.3).

We evaluated whether network estimates were sufficiently stable using bootstrap (Epskamp et al., 2018). In particular, we used the correlation stability coefficient (CS-coefficient), which allows for assessing the stability of node-level indices such as predictability and centrality. Cutoff values of 0.25 and 0.50, respectively, indicate sufficient and good stability (Epskamp et al., 2018). CS indicated high stability for all groups: F_ASSP (N = 2385): ranged from 0.61 to 0.75; F_Commerce (N = 1583): from 0.65 to 0.75; and M_MELEC (N = 1587): from 0.55 to 0.75, except M_Commerce (N = 1122), for which the CS was sufficient-to-high: from 0.43 to 0.75.

We compared differences in networks for females and males in the three educational tracks using a permutation approach (Van Borkulo et al., 2022). In terms of structure, the maximum absolute difference between two corresponding edges (M) is used, assessing network invariance. This measure indicates whether this specific edge has the same strength in both samples. A second comparison (S) tests whether the samples differ in the Global strength of the networks, indicated by the overall strength of connectivity, which is defined as the weighted absolute sum of all edges in a network.

All analyses were performed in the R statistical software package (version: 4.5.0; (R Core Team, 2017)). Descriptive statistics, reliabilities and correlations were computed using *psych* (Revelle, 2013) and *PerformanceAnalytics* (Peterson, 2020) packages. Networks were estimated using the packages *bootnet* and *qgraph* (Epskamp et al., 2012, 2018). To relax the normality assumption for network analysis, the nonparanormal transformation was applied with the R package *huge* (H. Liu et al., 2009; Zhao et al., 2012). We compared networks for females and males using the *NetworkComparisonTest* package (NCT; (Van Borkulo, 2016)). Clusters were inferred using the *iqgraph* package for R (Csárdi et al., 2025).

## 3. Results

### 3.1. Comparisons of the Absolute Values of the 28 Variables Among the Four Groups

**Figure 1:**
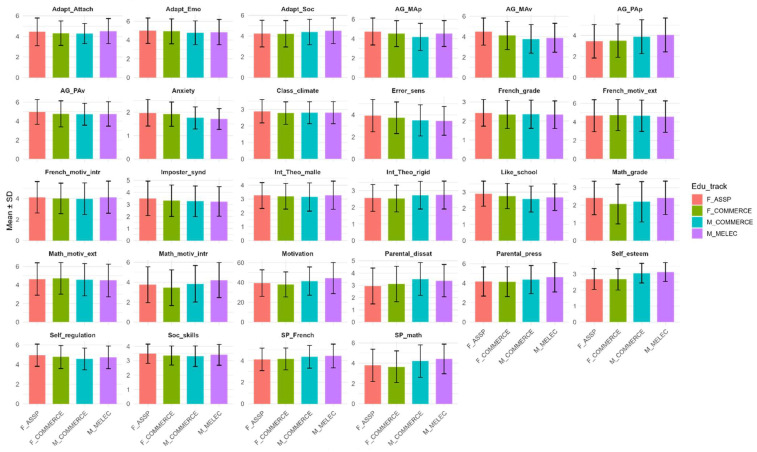
Means and SDs for 4 groups. Note: Like_school—To like school or not; Anxiety—School anxiety; Self-regulation—Self-regulation; AG_MAp—Approach Master Goals; AG_MAv—Avoidance Master Goals; AG_PAp—Approach Performance Goals; AG_PAv—Avoidance Performance Goals; Adapt_Emo—Emotional and personal adaptation; Adapt_Soc—Social adapting; Adapt_Attach—Attachment to the institution; Class_climate—Classroom climate; SP_French and SP_math—Self-perception in Math and French; Soc_skills—Social skills; Self_esteem—Global self-esteem; Motivation—Motivation for cognitive activities; French_motiv_ext and Math_motiv_ext—External motivation for math and French; French_motiv_intr and Math_motiv_intr—Intrinsic motivation for math and french; Parental_dissat—Perception of parental dissatisfaction; Parental_press—Parental pressure; Error_sens—Error sensitivity; Imposter_synd—Feeling like an academic imposter; Int_Theo_rigid—Theory of entity intelligence (rigid); Int_Theo_malle—Theory of incremental intelligence (malleable); French_grade and Math_grade—Math and French Competencies.

Descriptive statistics for males and females in different groups are presented in Figure 1.

Welch ANOVA comparisons (See Table S2) among the groups showed that the groups differed in all study variables, with effect sizes ranging from minuscule (e.g., for Theory of intelligence—malleable scale) to medium (e.g., self-esteem) as per conventional thresholds (Lakens, 2013; Richardson, 2011). Post hoc pairwise comparisons (Bonferroni-corrected) are available in Supplementary Materials Table S3. The largest differences were found between females in ASSP and males in Commerce for school anxiety (Cohen’s d = 0.40), whether to like school (0.42) and Mastery Avoidance (0.52); between females in ASSP and males in MELEC on school anxiety (0.51) and Mastery Avoidance (Cohen’s = 0.44); and between females in Commerce and males in MELEC on school anxiety (0.43), with females showing higher scores for these variables. Males had higher scores for self-esteem, with the largest differences found between females in Commerce and males in MELEC (−0.72), females in ASSP and males in MELEC (−0.69), females in Commerce and males in Commerce (−0.60) and females in ASSP and males in Commerce (−0.57).

As there were both males and females only for the Commerce track, we looked at comparisons within this track separately. Females demonstrated higher scores for school anxiety (Cohen’s d = 0.32), mastery approach (0.25), whether to like school (0.24) and Mastery Avoidance (0.24), and males—for self-esteem (−0.60) and self-perceived math competence (−0.35). To assess the effect of track only, we looked at differences between females in the ASSP and Commerce tracks. Females from ASSP demonstrated higher scores in math grades (0.33), Mastery Avoidance (0.29), social skills (0.19) and school enjoyment (0.19), and females in Commerce—higher scores in perceptions of parental dissatisfaction (−0.11), performance approach (−0.04) and External motivation in French (−0.04). However, the overall number of significant differences was small, and the effect sizes were, on average, smaller than those observed for gender. See Table S3 for Posthoc comparisons among 4 groups for all study variables.

### 3.2. Network Analysis for Anxiety-Related Variables in Four Groups

#### 3.2.1. Networks for the Four Groups

**Figure 2:**
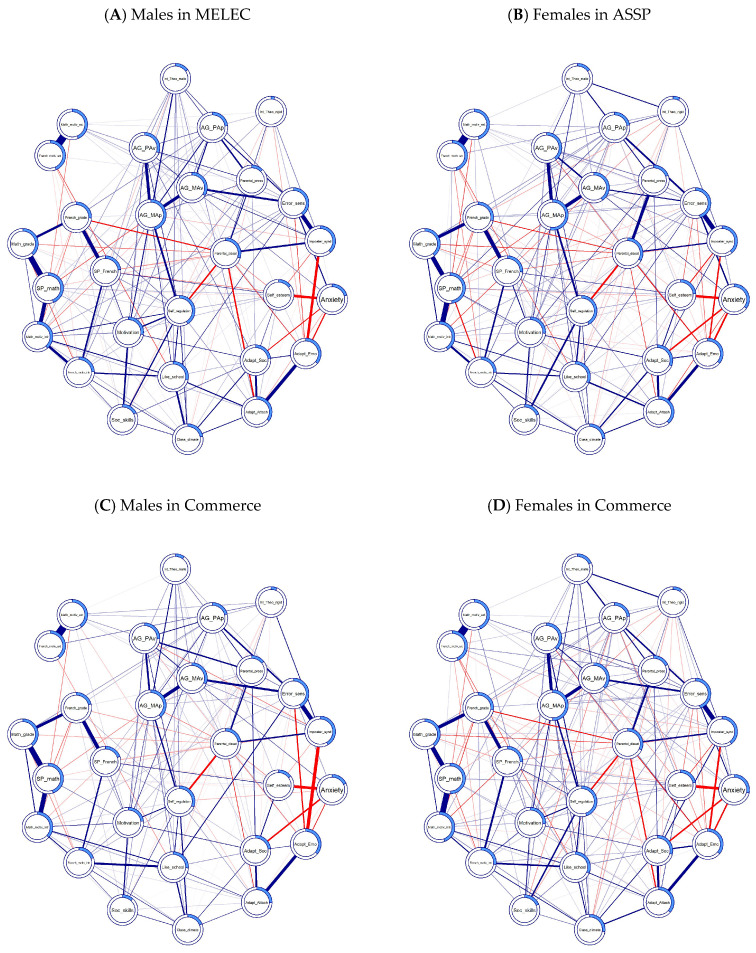
Networks for 4 groups. Note: Each node represents a variable; blue lines indicate positive connections; red lines indicate negative connections; the thickness of the lines represent the strength of the connection; the ring around the node indicates the predictability of the node; Like_school—To like school or not; Anxiety—School anxiety; Self-regulation—Self-regulation; AG_MAp—Approach Master Goals; AG_MAv—Avoidance Master Goals; AG_PAp—Approach Performance Goals; AG_PAv—Avoidance Performance Goals; Adapt_Emo—Emotional and personal adaptation; Adapt_Soc—Social adapting; Adapt_Attach—Attachment to the institution; Class_climate—Classroom climate; SP_French and SP_math—Self-perception in Math and French; Soc_skills—Social skills; Self_esteem—Global self-esteem; Motivation—Motivation for cognitive activities; French_motiv_ext and Math_motiv_ext—External motivation for math and French; French_motiv_intr and Math_motiv_intr—Intrinsic motivation for math and french; Parental_dissat—Perception of parental dissatisfaction; Parental_press—Parental pressure; Error_sens—Error sensitivity; Imposter_synd—Feeling like an academic imposter; Int_Theo_rigid—Theory of entity intelligence (rigid); Int_Theo_malle—Theory of incremental intelligence (malleable); French_grade and Math_grade—Math and French Competencies.

The network analysis yielded quite similar network structures for females and males in different tracks, with some differences in the magnitude of links across the groups (see Figure 2). For example, it seems that Imposter syndrome and emotional adaptation to school were more strongly connected in male compared to female samples, but anxiety was uniformly negatively linked to social and emotional school adaptation measures in all groups. Another example might be an overall stronger link between parental pressure and performance avoidance motivations in males compared to females. Also, there were, on average, more links in females’ networks compared to networks for males (see Table S4 in Supplementary Materials for the full list of network edges).

#### 3.2.2. Network Cluster Analysis

**Figure 3:**
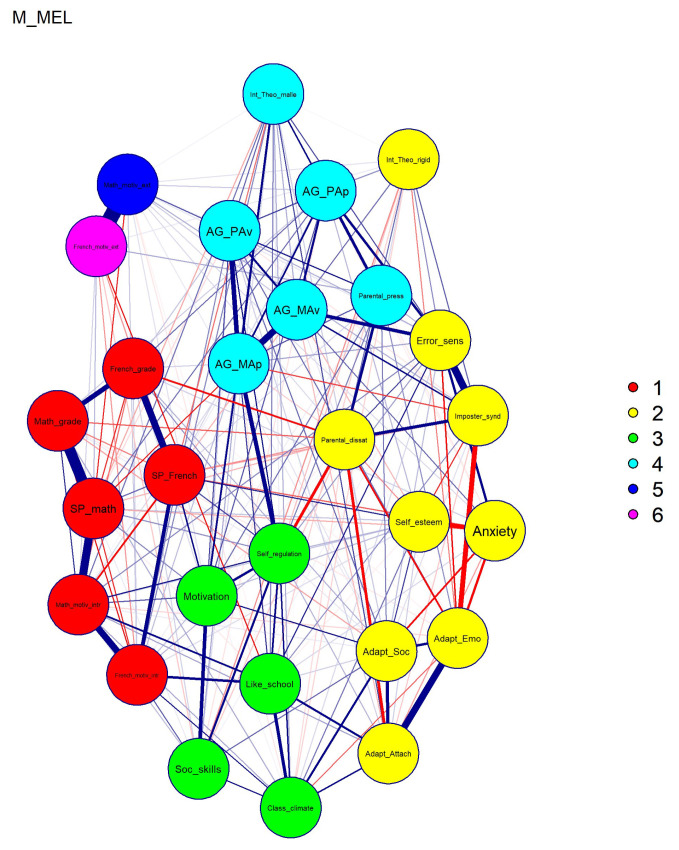
Cluster analysis for males in MELEC. Note: Each node represents a variable; blue lines indicate positive connections; red lines indicate negative connections; the thickness of the lines represents the strength of the connection; clusters are color-coded, with different clusters marked with different colors; Like_school—To like school or not; Anxiety—School anxiety; Self-regulation—Self-regulation; AG_MAp—Approach Master Goals; AG_MAv—Avoidance Master Goals; AG_PAp—Approach Performance Goals; AG_PAv—Avoidance Performance Goals; Adapt_Emo—Emotional and personal adaptation; Adapt_Soc—Social adapting; Adapt_Attach—Attachment to the institution; Class_climate—Classroom climate; SP_French and SP_math—Self-perception in Math and French; Soc_skills—Social skills; Self_esteem—Global self-esteem; Motivation—Motivation for cognitive activities; French_motiv_ext and Math_motiv_ext—External motivation for math and French; French_motiv_intr and Math_motiv_intr—Intrinsic motivation for math and french; Parental_dissat—Perception of parental dissatisfaction; Parental_press—Parental pressure; Error_sens—Error sensitivity; Imposter_synd—Feeling like an academic imposter; Int_Theo_rigid—Theory of entity intelligence (rigid); Int_Theo_malle—Theory of incremental intelligence (malleable); French_grade and Math_grade—Math and French Competencies.

Overall, network analysis showed a lot of links among 28 measures, with some of them forming relatively distinct clusters. Self-reported grades, self-perceived ability and intrinsic motivation for French and math formed a “Achievement and ability conceptions” cluster. Error sensitivity, Imposter syndrome, self-esteem, school anxiety, and three school adaptation measures formed a “Inhibitors of achievement” cluster. The third one included self-regulation, general motivation, social competence, school enjoyment and class climate—“Self-regulation” cluster. The fourth one included achievement goals, theory of intelligence and parental pressure—“Facilitators of achievement”. Two extrinsic motivation measures were linked with each other, but demonstrated weak links with other measures. See Figure 3 for cluster division of males in MELEC, with other graphs available in Supplementary Materials Figure S5. Bridge centrality metrics coming from cluster analysis are presented in Supplementary Materials Figure S6; the most important nodes according to Bridge Expected Influence are presented in Table 2.

#### 3.2.3. Network Centrality

The mastery approach achievement goals scale was the most important node according to the Expected Influence metric for all four groups. Anxiety demonstrated the lowest EI for three out of four groups. Other centrality indices showed some differences among groups (See Table 2). Strength centrality also demonstrated mastery approach being important for males in Commerce and MELEC groups. For females, it was self-reported math competence in the ASSP group and self-regulation in the Commerce groups. Figure S7 in Supplementary Materials presents centrality metrics for all nodes in four networks.

**Table 2:** Nodes with the highest centrality according to different metrics in 4 groups.

	Definition	F_ASSP	F_COMMERCE	M_COMMERCE	M_MELEC
Strength	Node importance and potential influence on other nodes	Self-reported math competence	Self-regulation	Mastery approach	Mastery approach
Highest Expected influence	Extension of Strength that demonstrate node importance but accounts for positive and negative links	Mastery approach	Mastery approach	Mastery approach	Mastery approach
Lowest Expected influence		Anxiety	Parental dissatisfaction/Anxiety	Anxiety	Anxiety
Bridge centrality: Expected influence	Greatest potential to influence multiple network clusters	Error Sensitivity	Error Sensitivity	Mastery approach	Self-regulation
Node predictability	How well the node is explained by other nodes in the network	Mastery approach	Mastery approach	Self-reported math competence	Mastery approach

We repeated the network analysis without three measures that demonstrated lower reliability and got similar results in terms of links and centrality metrics distribution (though the metrics of networks’ stability were slightly higher). These results are available in Supplementary Materials Figures S8 and S9.

Additionally, we conducted network analysis for the males in the ASSP group (N = 170)—the one that was excluded from the main analysis, but failed to produce a reliable model (CS coefficients were below the recommended 0.25 level; see Figure S4 for network graph in Supplementary Materials).

#### 3.2.4. Network Comparison Test Across Four Groups

Table 3 presents network comparison tests among the groups. Overall, global strength (S) results suggest that the psychological constructs in female groups were more tightly interconnected (i.e., demonstrated stronger mutual associations) compared to male ones. Network invariance test (M) showed that at least some edges differ in magnitude or presence for different groups.

## 4. Discussion

In the current study, we explored complex links among 28 anxiety-related constructs and identified clusters of traits and bridge symptoms that connect them. Although quite similar network patterns emerged across the four groups, our data also revealed differences in absolute values and network structures between males and females with different educational backgrounds.

### 4.1. Absolute Differences Across Four Groups in the Study Variables

Our data showed some differences of small-to-moderate size among the study samples. The largest differences were found between males and females (rather than among educational tracks), with the data in general converging with previous research. For example, our data showed higher self-confidence in males, which is in line with multiple studies on general self-confidence and in specific fields, such as creativity and mathematics (Bleeker & Jacobs, 2004; Hofer et al., 2025; Preckel et al., 2008; Rammstedt & Rammsayer, 2002; Repeykova et al., 2023), and in math performance, which is also a widely replicated effect (Geary et al., 2023; M. Likhanov et al., 2024a; Lu et al., 2023; Toivainen et al., 2017). Conversely, females showed higher scores for anxiety and greater error sensitivity, which replicates multiple previous studies (Burani & Nelson, 2020; Chaplin & Aldao, 2013; McLean et al., 2011; Panayiotou et al., 2017; Strand et al., 2021). Importantly, females showed more endorsement for mastery avoidance and mastery approach achievement goals, which is in line with previous studies that showed females are more inclined to mastery profiles, while males demonstrate a more mixed pattern (Butler, 2014; Fiévé et al., 2025; Schwinger et al., 2016).

### 4.2. Connections Among the Nodes and Cluster Analysis—Theoretical Implications

We identified four clusters which were more readily visible in males compared to females. For example, in female groups, there were three clusters, rather than four, with one cluster (“Self-regulation” and “Facilitators of achievement” were clashed) being pressed by “Inhibitors of achievement” (one that included anxiety and error sensitivity) and “Achievement and ability conceptions” (one that included achievement and motivation) clusters on both sides. This could be explained by the greater interconnectedness in female groups, as reflected in Global strength indices. Such interconnectedness may indicate a more tightly coupled network structure, reflected in stronger associations among nodes (Borsboom, 2017). Although prior theoretical and simulation work has suggested that higher connectivity may be associated with increased susceptibility to cascading activation or reduced flexibility in response to stress, these interpretations remain tentative and cannot be directly inferred from the present cross-sectional analyses (Cramer et al., 2016; Robinaugh et al., 2020). More generally, greater connectivity has been linked to stronger co-occurrence among traits, such that tightly interconnected adaptive traits may be associated with better functioning, whereas tightly interconnected risk-related traits may be associated with greater vulnerability (e.g., anxiety; Robinaugh et al., 2020; Schueler et al., 2021).

In addition, some nodes were shown to demonstrate different affiliations to clusters in different samples. For example, perceptions of parental dissatisfaction belong to the anxiety-related “Inhibitors of achievement” cluster in males, but to a large mixed cluster in females. It is possible that this “shifting” affiliation stems from this node being a hub or a mediatory pathway between the clusters (relatively high on Bridge centrality), which could be “gatekeepers” for interventional effects (Epskamp et al., 2018; Harder et al., 2025). Specifically, parental dissatisfaction could inhibit the effects of a “negative” cluster on the “positive” one. And indeed, previous randomized control trials showed that perceptions of parental dissatisfaction could positively affect the overall well-being of a student by precluding the negative effects of stressors (Kallianta et al., 2021; Yeager et al., 2022). These findings suggest that helping adolescents reframe academic challenges (mindset/coping interventions) and fostering supportive parent–child dialog (which could be especially important for VET students; e.g., (Abela & Camilleri, 2024)) can mitigate the distress associated with feeling one’s parents are unhappy about school performance.

The current study may also help to plan future observational and experimental studies, as the identified links could help to formulate predictions regarding connections among different traits. For example, previous studies mostly used one variable as mediator of the link between achievement and anxiety (e.g., self-esteem; (Van Der Beek et al., 2017; Zhang et al., 2023) or parenting styles; (Albulescu et al., 2023)), while our results suggest that other longer paths may exist via error sensitivity, Imposter syndrome and perceptions of parental dissatisfaction or via school adaption measures and school enjoyment. These paths also seem plausible as per contemporary literature (Harris et al., 2023; Leenknecht et al., 2019) and require further experimental and longitudinal research to be confirmed.

### 4.3. Network Centrality Indices—Practical Implications

Our data showed that the most important traits in the network according to Strength and Expected Influence centrality metrics—potential targets for educational interventions (see Bringmann et al., 2019; Robinaugh et al., 2016)—varied for different groups.

#### 4.3.1. Mastery Approach Goals Are Central for All Groups

Mastery approach achievement goals were shown to be central according to the EI centrality metric in all four groups, and according to Strength for males in both the MELEC and Commerce groups. As mastery approach motivations are associated with the use of deep learning strategies, enhanced intrinsic motivation and greater persistence in the face of academic challenges, and higher academic achievement (Kaplan et al., 2002; Midgley et al., 2001), and were shown to be malleable through different approaches, including interactive self- and peer-assessment activities (Yokoyama & Miwa, 2021); promotion of mastery goal or task goal orientation in six TARGET areas (Cecchini-Estrada & Méndez-Giménez, 2017); and encouraging a mastery focus in order to reduce stereotype threat (Canning et al., 2019; Good et al., 2003)—endorsement of these goals could be a valuable target for educational interventions.

It should be noted, though, that performance approach goals are usually more readily linked to performance compared to mastery approach goals (see, e.g., a meta-analysis; Hulleman et al., 2010). In our data, mastery approach goals indeed showed no direct links to performance, but are directly linked to self-regulation, which is linked to performance via motivation, suggesting a complex mechanism of potential intervention effects. Interestingly, perceptions of parental pressures belonged to the same cluster as achievement goals (which is in line with some previous studies that demonstrated correlations between them; (Fieve et al., 2025; Xiang et al., 2017)), suggesting that future interventions targeting achievement goals should also account for parents as an important factor.

#### 4.3.2. Self-Perceived Math Competence for Females in the ASSP Group

Self-perceived math competence was shown to be central as per the strength centrality index for females in the ASSP group. A number of explanations could be put forward for why this particular trait is important for females in an educational track that is not directly related to math or STEM (unlike, e.g., Commerce), and for which they showed the lowest score among the four groups. One primary explanation could be that females perceive ASSP as a “resort from math” (see, e.g., Megreya & Al-Emadi, 2023), due to various reasons, including teacher expectations (Martinot et al., 2025; Trusz, 2020), stereotypes (Bauer & Job, 2024; Repeykova et al., 2023), underestimation of their math ability (Stoet & Geary, 2018) and higher math anxiety at previous levels of education (Eidlin-Levy et al., 2023; Ferdinand et al., 2024). Females from ASSP do not select mathematics as their major but may still experience pressure in relation to it, as both societies and governments put a lot of stress on it (see, e.g., some regulations in France; *21 Measures for Teaching Mathematics*, 2018; *National Reforms in School Education*, 2025). The Usage of Expected Influence metric instead of Strength showed that when the negative connections of self-perceived math competence (a reflection of maths avoidance in this sample—only those with low maths achievement/self-perceived maths ability landed in VET schools) were accounted for, mastery approach motivations became the most central node.

At the same time, confidence in math is vital in caring and teaching-support professions (e.g., assisting pupils with disabilities in primary or secondary schools). Women dominate fields like nursing, primary education and early childcare in France and across Europe, yet many report low math self-efficacy. For instance, one study showed that nurses who failed drug dosage calculation tests were significantly more anxious and less confident about math than those who passed (McMullan et al., 2012). Similarly, pre-service teachers often exhibit high math anxiety and low self-efficacy. For example, a study of secondary teacher trainees reported relatively high math anxiety, which was especially high among female students (Boateng et al., 2025). Such anxiety undermines teachers’ confidence in teaching math effectively and could transfer to their female students (Beilock et al., 2010). To sum up, educational stakeholders (teachers, governmental employees, etc.) may target self-perceived math competence in females in order to improve their overall achievement and well-being (Samuel & Warner, 2021; Zakariya, 2022).

#### 4.3.3. Self-Regulation for Females in Commerce Groups

Self-regulation—the capacity to concentrate on tasks, organize one’s work, and study autonomously—was demonstrated as being important for females in the Commerce educational track as per the Strength centrality index. A potential explanation for this might be that this trait conceptually overlaps with Big Five Conscientiousness (from definition; (Furnham et al., 2009)), which was shown to be the strongest (out of the five traits) predictor of academic achievement in unselected samples (Noftle & Robins, 2007; Poropat, 2009). Further, conscientiousness is positively correlated with performance in exams, essays, continuous assessment and supervised dissertations (Furnham et al., 2009; O’Connor & Paunonen, 2007; Papageorgiou et al., 2020).

Research also shows that females, on average, demonstrate more conscientiousness than males (Hofmann et al., 2025; M. V. Likhanov et al., 2021; E. S. Tsigeman et al., 2025). Further, one study reported that women generally score higher than men on facets of conscientiousness, such as orderliness and self-discipline (Weisberg et al., 2011)—ones that are tapped into by the self-regulation questionnaire used in the current study. Females also scored higher on this trait in the current study. It is possible that females rely on their ability to plan and focus on the task at hand in order to succeed in Commerce schools. And indeed, research shows girls often employ more self-regulatory learning strategies than boys (evidence from VET: (Rozendaal et al., 2003)), and that girls’ superior self-control at least partly mediated their higher grades relative to boys (Kling et al., 2013). It should be noted that self-regulation is strongly negatively directly linked to parental dissatisfaction (and positively to other nodes), which could explain why it was the most influential according to Strength but less important according to the EI index (that accounted for positive vs. negative associations).

### 4.4. Limitations and Future Directions

Firstly, we used an analytical approach that does not allow for drawing causal predictions (directionality of effects) from the data. Thus, suggested directions of effects are based on previous longitudinal and experimental studies. Secondly, translational studies (see discussion in Costantini & Perugini, 2018) are needed to assess whether interventions that target central nodes indeed lead to improvement in other nodes of the anxiety-related network. In any case, interventions targeting specific nodes are likely to primarily influence proximal nodes (e.g., within the same cluster), while having limited effects on more distal nodes. Thirdly, reliability for some measures was low (External motivation for math and French, and Rigid facet of Theory of intelligence), which could have affected the connectedness of the respective nodes to other nodes in the network (De Ron et al., 2022) and could have masked their role in the network. Measurement error associated with low reliability can attenuate correlations, leading to weaker or unstable edges and, consequently, reduced centrality estimates for the affected nodes. Moreover, unreliable measures may distort the overall network structure by obscuring true associations or introducing spurious patterns, thereby limiting the interpretability and replicability of the estimated network (Fried & Cramer, 2017)—which was not the case for the current study, as exclusion of these variables demonstrated similar network structures. Alternatively, low centrality for these variables may confirm previous research that showed the little role of conceptions of intelligence in achievement (see, e.g., a meta-analysis; Macnamara & Burgoyne, 2023). In addition, this study relied on self-reported measures of personality (such as for motivation and anxiety) and related constructs, which have a number of limitations (see for discussion, E. Tsigeman et al., 2023); future research could benefit from using standardized cognitive tests (e.g., for math performance) or national records to better assess the constructs of interest. Fourthly, the current study lacked data from general schools, which did not allow us to compare the results obtained in VET with the general educational track. For example, we cannot compare whether anxiety was truly higher in our VET samples compared to the general population of schoolchildren. Sixthly, gender imbalance in ASSP (mostly females) and MELEC (mostly males) schools precluded gender by track investigation. We excluded males from ASSP and females from MELEC due to their small samples, making comparisons between males and females in the Commerce track the only “clean” gender difference comparisons. Finally, deletion of outliers could have masked some effects of anxiety on other traits, as we deleted “extreme” cases. However, we consider our results still valid as the anxiety distributions were positively skewed even before deleting outliers—similarly to other research in nonclinical populations (e.g., M. Likhanov et al., 2024a).

## 5. Conclusions

To sum up, the current study has made four significant theoretical contributions. First, we showed that the links within this large network of anxiety-related traits are complex, with variables forming four clusters: Achievement and ability conceptions, Inhibitors of achievement, Self-regulation and Facilitators of achievement. Second, we identified differences in links, centrality index distributions and clusters of nodes across the studied groups. The observed differences were relatively small and were driven primarily by gender and, to a lesser extent, by educational track. Third, we showed that mastery approach goals demonstrate the highest centrality according to the Expected Influence metric and, thus, may be targeted by educational interventions if there are no resources to implement different interventions for specific groups. Where feasible, the more targeted approach could be implemented with self-reported math competence targeted in females from the ASSP group, self-regulation in females from Commerce, and mastery approach goals in males for both MELEC and Commerce groups—nodes highest on Strength centrality. Fourth, we showed that the links between two variables may be more complex compared to how we usually frame them, with multiple variables potentially acting as mediators of effects. For example, the path from anxiety to maths performance might not be direct and is mediated by multiple school, family and personal variables. Taken together, these results provide a basis for translational research and may help to make predictions about mechanisms of links among different variables and effects of interventions.

## Figures and Tables

**Table 1:** Description of study variables.

Variable Name	Construct	N Items	Example Item	Reliability in the Current Sample	Reference
Like_school	To like school or not	3	How much do you like school?	0.71	Developed by Authors, validation paper is in preparation
Anxiety	School anxiety	10	Usually at school or before going there I feel embarrassed around teachers or other students	0.84	(Pouille, 2016)
Self-regulation	Self-regulation	9	I do my work and homework without my parents behind me	0.90	Adapted from (Pintrich et al., 1991)
AG_MAp	Mastery Approach Goals	3	I want to have complete control over the content of my courses	0.86	(Elliot & McGregor, 2011) Adapted in: (Darnon & Butera, 2005)
AG_MAv	Mastery Avoidance Goals	3	I’m worried that I won’t learn as much as I could in my courses	0.82	
AG_PAp	Performance Approach Goals	3	It’s important for me to do better than other students	0.89	
AG_PAv	Performance Avoidance Goals	3	I just don’t want to fail my courses	0.77	
Adapt_Emo	Emotional and personal adaptation	5	What I miss here is someone with whom I can chat freely from time to time	0.81	Adapted from (Dahmus et al., 1992)
Adapt_Soc	Social adapting	5	I really feel at home here at this school	0.75	
Adapt_Attach	Attachment to the institution	5	I’m glad I came here to study	0.71	
Class_climate	Classroom climate	14	Autonomy scale: In the classroom, my teacher lets me choose certain things (like which texts to work on, which books to read, when to do which things, etc.). Competence scale: My teacher checks that I’ve really understood what needs to be done during the class. Affiliation scale: I feel that my teacher understands me	Autonomy: 0.72 Competence: 0.67 Affiliation: 0.78	(Williams & Deci, 1996)
SP_French	Self-perception in Math and French	6	I feel as good as other students my age in French/Math	FR 0.76	Adapted from (Harter, 1985) and validated in (Núñez-Regueiro et al., 2022, 2026)
SP_math		6		Math 0.88	
Soc_skills	Assessment of social skills in adolescence	8	I avoid making others look bad	0.72	Translated into French from Social competence teen survey: measure developed by Child Trends for the Flourishing Children Project, funded by the Templeton Foundation (www.childtrends.org).
Self_esteem	Global self-esteem	5	Some young people are often disappointed with themselves BUT other young people are fairly satisfied with themselves	0.80	(Bariaud, 2006)
Motivation	Motivation for cognitive activities	32	The questionnaire included 8 cognitive activities (e.g., mathematics), with frequency, intrinsic (e.g., I like it), extrinsic (I’m forced to do it) and identified (e.g., It’s useful) motivation being assess to each.	0.84	Authors—in review
French_motiv_ext	External motivation for math and French	2	I don’t want my teacher to argue with me	fr 0.12 *	(Ryan & Connell, 1989)
Math_motiv_ext		2		Math 0.12 *	
French_motiv_intr	Intrinsic motivation for math and French	2	It’s fun doing what we do	fr 0.66	
Math_motiv_intr		2		Math 0.91	
Parental_dissat	Perception of parental dissatisfaction	4	My parents are dissatisfied with my efforts at school	0.79	Items developed by the Unité de recherche sur l’affectivité, la motivation et l’apprentissage scolaires (URAMAS), Université du Québec à Montréal.
Parental_press	Parental pressure	4	My parents push me to get the best grades possible	0.79	
Error_sens	Error sensitivity	7	I think that if I make a mistake, it’s as if all my work is bad	0.87	(Seidah et al., 2002)
Imposter_synd	Feeling like an academic imposter	8	I think it will eventually become apparent that I’m not as good as others think	0.89	(Bouffard et al., 2005)
Int_Theo_rigid	Theory of entity intelligence (rigid)	3	To be intelligent, you need to have certain qualities from birth	0.51	(Da Fonseca et al., 2004)
Int_Theo_malle	Theory of incremental intelligence (malleable)	3	You have to work hard to be intelligent”	0.74	
French_grade	Self-reported Math and French Competencies	1	From memory, what was your average in French/Math at the end of the last school year?	NA	Questions were developed by: (Janosz et al., 2007)
Math_grade		1			

Note: the reliability was computed using Cronbach’s Alpha; * for external and internal motivation, a Spearman–Brown reliability coefficient was used, as it is better suited to two-item scales (Eisinga et al., 2013).

**Table 3:** Network comparison tests.

	Global Strength (S)			Network Invariance Test (M)		
Track (S ^#^)	M_COMMERCE (12.66)	M_MELEC (12.97)	F_ASSP (14.92)	M_COMMERCE	M_MELEC	F_ASSP
M_MELEC (12.97)	0.31	0		0.11	0	
F_ASSP (14.92)	2.25	1.94	0	0.13 *	0.17 ***	0
F_COMMERCE (14.59)	1.93 *	1.62 *	0.33	0.13 *	0.13 **	0.08

Note: * *p* < 0.05; ** *p* < 0.01, and *** *p* < 0.001; ^#^ Global strength indices are presented in brackets for each group.

## Data Availability

The R code for the analyses presented here is publicly accessible at https://osf.io/bp9wy/?view_only=64e7bbf2459f4b699a90e6060946fa55 (accessed on 10 May 2025). The data belongs to the ProFan project and could not be freely shared. All inquiries regarding access to the data should be addressed to marie.demolliens@uca.fr. The analyses presented here were not pre-registered.

## Supplementary Materials

The following supporting information can be downloaded at: https://www.mdpi.com/article/10.3390/bs16050706/s1. Table S1: Age frequency distributions for the 4 groups. Table S2: Welch ANOVA results for study variables. Table S3: Posthoc comparisons among 4 groups for all study variables. Table S4: Network edges for 4 groups. Figure S1: Missing data diagnostics and pattern of data missingness. Figure S2: Number of outliers. Figure S3: Distributions for all study variables divided by track. Figure S4: Network plot and centrality indices for Males is ASSP group. Figure S5: Cluster division for the four groups. Figure S6: Bridge centrality for cluster analysis. Figure S7: Full lust of centrality indices for EBIC-glasso network. Figure S8: Network plots with Rigid Theory of intelligence and external motivation in French and maths excluded. Figure S9: Centrality plots with Rigid Theory of intelligence and external motivation in French and maths excluded.

## Appendix A

ProFAN consortium members:

- **Batruch Anatolia**, Laboratoire de Psychologie Sociale de l’Université de Lausanne—UnilaPS Université Lausanne, Lausanne, Switzerland & LIVES Centre, University of Lausanne, Lausanne, Switzerland
- **Bouet Marinette**, Université Clermont Auvergne, CNRS, LIMOS, F-63000 Clermont-Ferrand, France
- **Bressoux Pascal**, Laboratoire de Recherche sur les Apprentissages en Contexte—LaRAC EA602 Université Grenoble, Grenoble, France
- **Brown Genavee**, Univ Rennes, LP3C (Laboratoire de Psychologie: Cognition, Comportement, Communication)—F-35000 Rennes, France & Northumbria University, Newcastle, UK
- **Butera Fabrizio**, Laboratoire de Psychologie Sociale de l’Université de Lausanne—UnilaPS Université Lausanne, Lausanne, Switzerland
- **Cherbonnier Anthony**, Univ Rennes, LP3C (Laboratoire de Psychologie: Cognition, Comportement, Communication)—F-35000 Rennes, France & Centre interuniversitaire de recherche en éducation de Lille, Lille, France
- **Darnon Céline**, Université Clermont Auvergne, CNRS, LAPSCO, F-63000 Clermont-Ferrand, France
- **Demolliens Marie**, Université Clermont Auvergne, CNRS, LAPSCO, F-63000 Clermont-Ferrand, France
- **De Place Anne-Laure**, Laboratoire de Recherche sur les Apprentissages en Contexte—LaRAC EA602 Université Grenoble, Grenoble, France & Université Paris 8, Saint-Denis, France
- **Desrichard Olivier**, Groupe de recherche en psychologie de la santé, GREPS, Université de Genève, Genève, Switzerland
- **Fillon Adrien**, Université Clermont Auvergne, CNRS, LAPSCO, F-63000 Clermont-Ferrand, France
- **Goron Luc**, Univ Rennes, LP3C (Laboratoire de Psychologie: Cognition, Comportement, Communication)—F-35000 Rennes, France
- **Huguet Pascal**, Université Clermont Auvergne, CNRS, LAPSCO, F-63000 Clermont-Ferrand, France
- **Jamet Éric**, Univ Rennes, LP3C (Laboratoire de Psychologie: Cognition, Comportement, Communication)—F-35000 Rennes, France
- **Likhanov Maxim**, Aix Marseille Univ, CNRS, CRPN, Marseille, France
- **Mazenod Vincent**, Université Clermont Auvergne, CNRS, LIMOS, F-63000 Clermont-Ferrand, France
- **Mella Nathalie**, Faculté de psychologie et des sciences de l’éducation, Université de Genève, Genève, Suisse & Centre Interfacultaire en sciences affectives, Université de Genève, Genève, Suisse
- **Michinov Estelle**, Univ Rennes, LP3C (Laboratoire de Psychologie: Cognition, Comportement, Communication)—F-35000 Rennes, France
- **Michinov Nicolas**, Univ Rennes, LP3C (Laboratoire de Psychologie: Cognition, Comportement, Communication)—F-35000 Rennes, France
- **Núñez-Regueiro Fernando**, Laboratoire de Recherche sur les Apprentissages en Contexte—LaRAC EA602 Université Grenoble, Grenoble, France
- **Ofosu Nana**, Groupe de recherche en psychologie de la santé, GREPS, Université de Genève, Genève, Switzerland
- **Pansu Pascal**, Laboratoire de Recherche sur les Apprentissages en Contexte—LaRAC EA602 Université Grenoble, Grenoble, France
- **Peter Laurine**, Univ Rennes, LP3C (Laboratoire de Psychologie: Cognition, Comportement, Communication)—F-35000 Rennes, France
- **Poletti Céline**, Aix Marseille Univ, CNRS, CRPN, Marseille, France & Institut de Psychologie, Université de Lausanne, Lausanne, Switzerland
- **Régner Isabelle**, Aix Marseille Univ, CNRS, CRPN, Marseille, France
- **Riant Mathilde**, Laboratoire de Recherche sur les Apprentissages en Contexte—LaRAC EA602 Université Grenoble, Grenoble, France
- **Robert Anais**, Université Clermont Auvergne, CNRS, LAPSCO, F-63000 Clermont-Ferrand, France
- **Rudmann Ocyna**, Laboratoire de Psychologie Sociale de l’Université de Lausanne—UnilaPS Université Lausanne & GREPS, Université de Genève, Genève, Switzerland
- **Toumani Farouk**, Université Clermont Auvergne, CNRS, LIMOS, F-63000 Clermont-Ferrand, France
- **Visintin Emilio Paolo**, Laboratoire de Psychologie Sociale de l’Université de Lausanne—UnilaPS Université Lausanne & Département de Sciences Humaines, Université de Ferrara, Ferrara, Italie
- **Vives Eva**, Aix Marseille Univ, CNRS, CRPN, Marseille, France & Moral & Social Brain Lab, Department of Experimental Psychology, Ghent University, Ghent, Belgium
