# Supplementary material for: Leverage Points for Wellbeing and Achievement in Vocational Education: A Network Analysis of Psychological Factors Across Gender and Majors
Source: Behav Sci (Basel). 2026 May 5;16(5):706. doi: 10.3390/bs16050706 (PMC13203397; doi:10.3390/bs16050706)
Supplement: Supplementary file 1 [file behavsci-16-00706-s001.zip › behavsci-4238399-supplementary-update.pdf]

Supplementary online materials for Leverage Points for Wellbeing and Achievement in Vocational Education: A Network Analysis of Psychological Factors Across Gender and Majors

Table S1 Age frequency distributions for the 4 groups

Table S1 Frequency for age divided by group x sex variable

	Age	Frequency	Percent
F_ASSP	less than 15	3	0.098
	15	233	7.592
	16	1571	51.189
	17	811	26.426
	18	106	3.454
	19	29	0.945
	20 and more	9	0.293
	Total	3069	100.000
F_COMMERCE	less than 15	4	0.190

Table S1 Frequency for age divided by group x sex variable

	Age	Frequency	Percent
	15	152	7.211
	16	1056	50.095
	17	506	24.004
	18	107	5.076
	19	18	0.854
	20 and more	12	0.569
	Total	2108	100.000
M_COMMERCE	less than 15	6	0.339
	15	104	5.869
	16	743	41.930
	17	474	26.749
	18	114	6.433
	19	28	1.580
	20 and more	28	1.580
	Total	1772	100.000

Table S1 Frequency for age divided by group x sex variable

	Age	Frequency	Percent
M_MELEC	less than 15	5	0.221
	15	121	5.349
	16	1077	47.613
	17	622	27.498
	18	130	5.747
	19	31	1.370
	20 and more	45	1.989
	Total	2262	100.000

Figure S1 Missing data diagnostics and pattern of data missingness

A

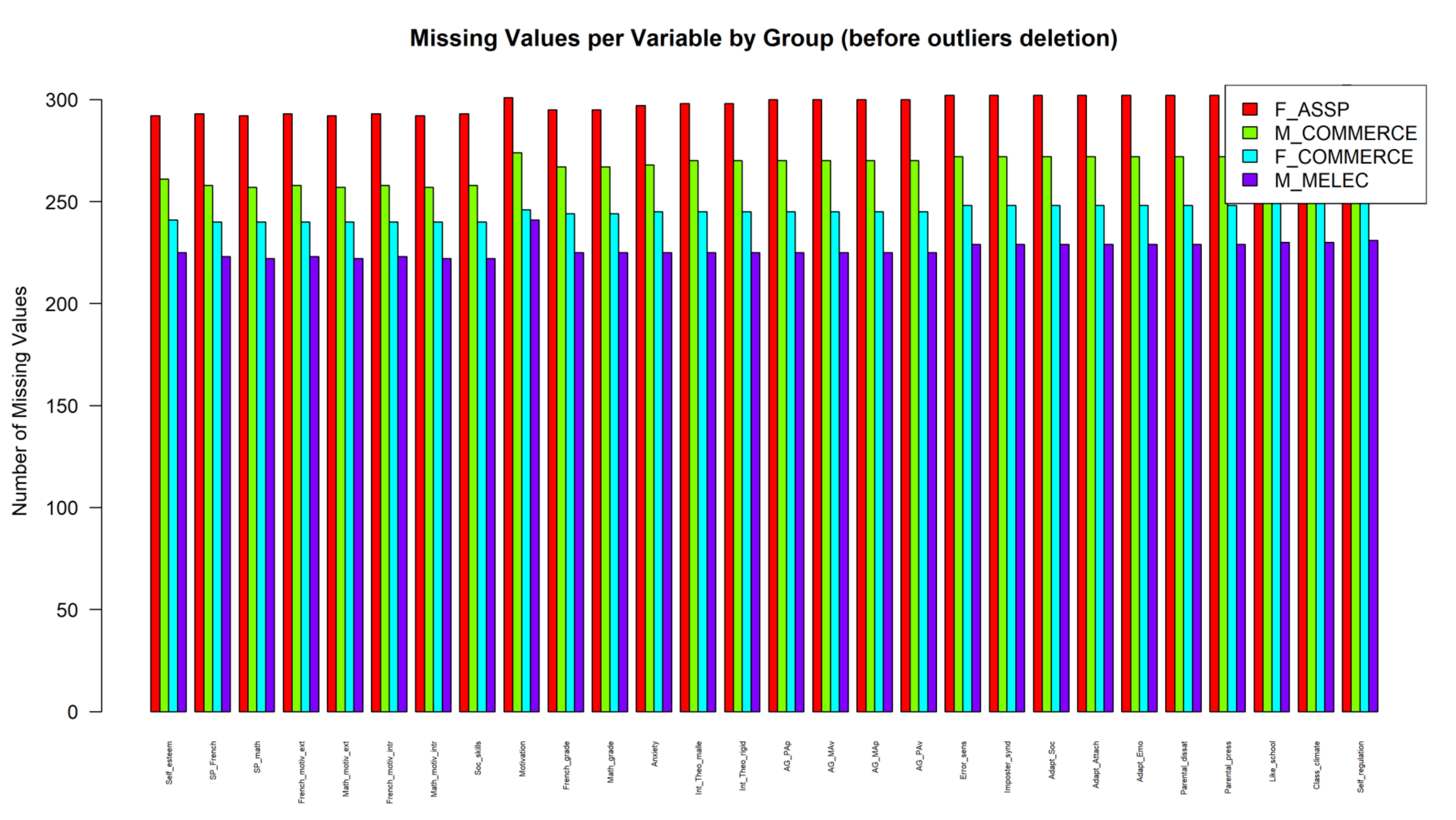

B

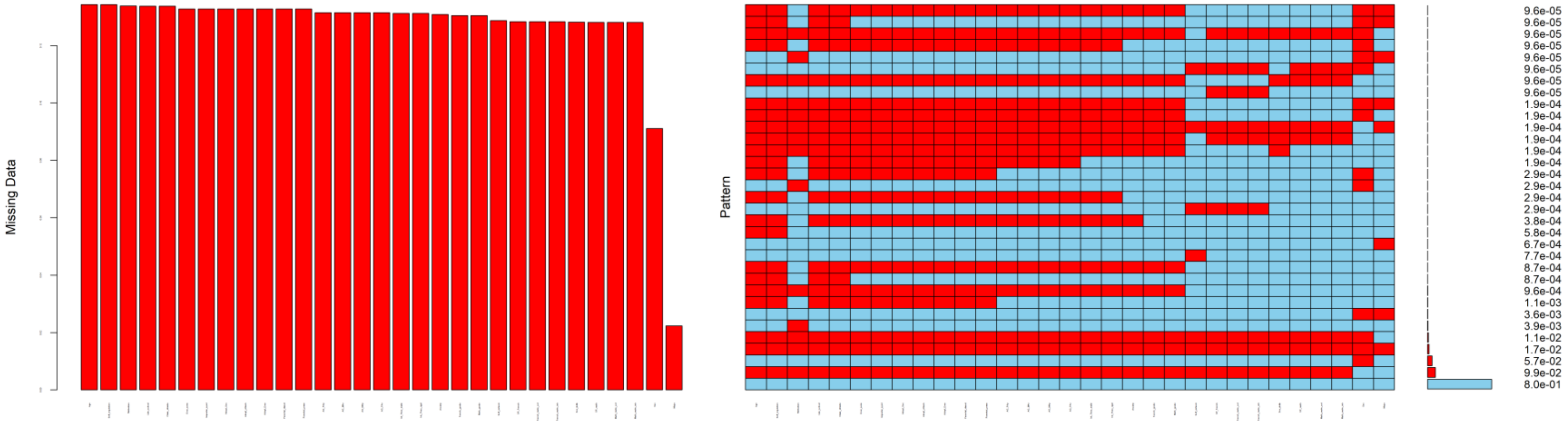

Missing Data
Pattern
9.6e-05
9.6e-05
9.6e-05
9.6e-05
9.6e-05
9.6e-05
9.6e-05
9.6e-05
1.9e-04
1.9e-04
1.9e-04
1.9e-04
1.9e-04
1.9e-04
2.9e-04
2.9e-04
2.9e-04
2.9e-04
3.8e-04
5.8e-04
6.7e-04
7.7e-04
8.7e-04
8.7e-04
9.6e-04
1.1e-03
3.6e-03
3.9e-03
1.1e-02
1.7e-02
5.7e-02
9.9e-02
8.0e-01

C

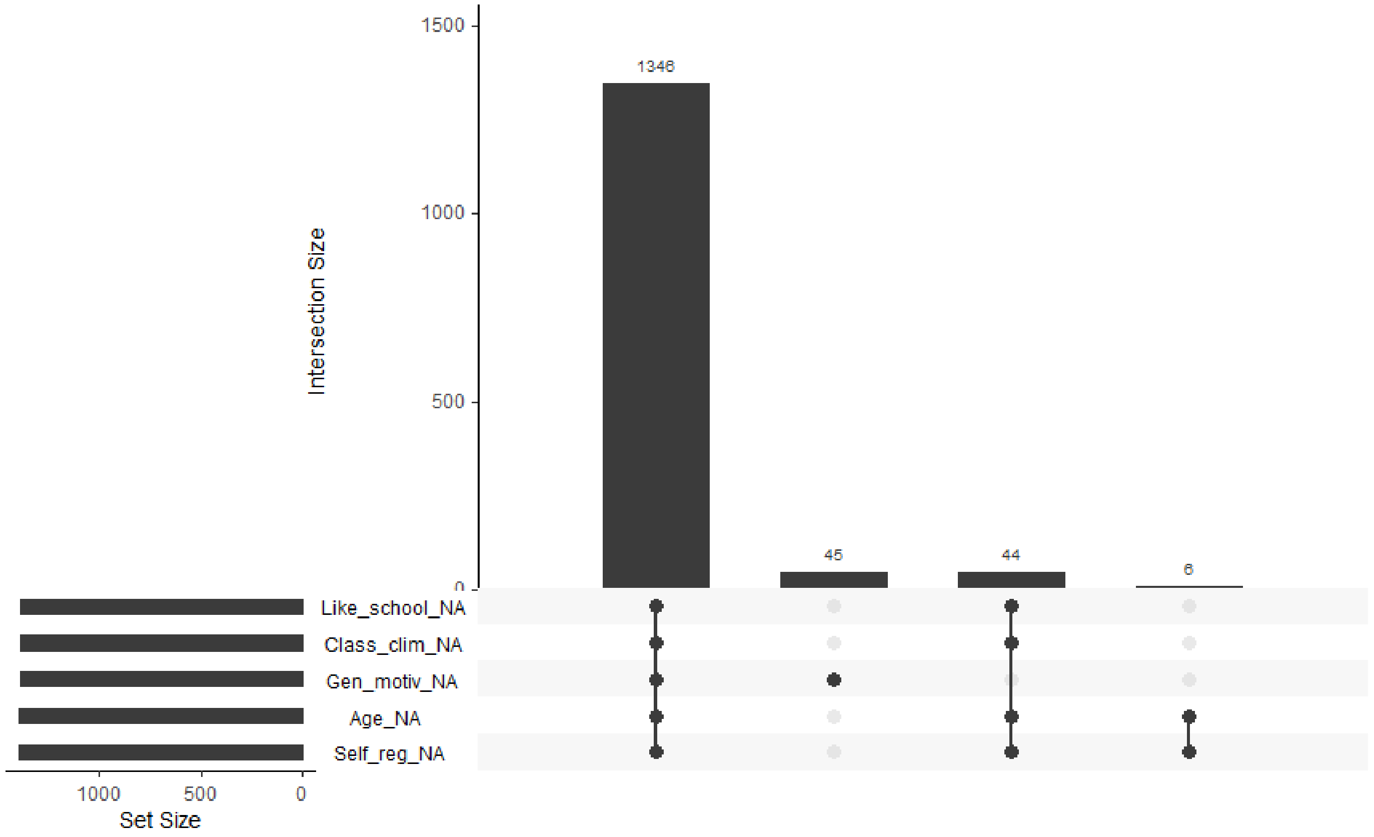

Note: Like_school – To like school or not; Anxiety – School anxiety; Self-regulation – Self-regulation; AG_MAp – Approach Master Goals; AG_MAv – Avoidance Master Goals; AG_PAp – Approach Performance Goals; AG_PAv – Avoidance Performance Goals; Emo_adapt – Emotional and personal adaptation; Soc_adapt – Social adapting; Attach_adapt – Attachment to the institution; Class_climate – Classroom climate; SP_French and SP_math– Self-perception in Maths and French; Soc_skills – Social skills; Self_esteem – Global self-esteem; Motivation – Motivation for cognitive activities; French_motiv_ext and Math_motiv_ext – External motivation for maths and French; French_motiv_intr and Math_motiv_intr – Intrinsic motivation for maths and french; Parental_dissat – Perception of parental dissatisfaction; Parental_press – Parental pressure; Error_sens – Error sensitivity; Imposter_synd – Feeling like an academic imposter; Int_Theo_rigid – Theory of entity intelligence (rigid); Int_Theo_malle – Theory of incremental intelligence (malleable); French_grade and Math_grade – Maths and French Competencies

Figure S2 Number of outliers

Figure S2 Number of outliers in each variable divided by group

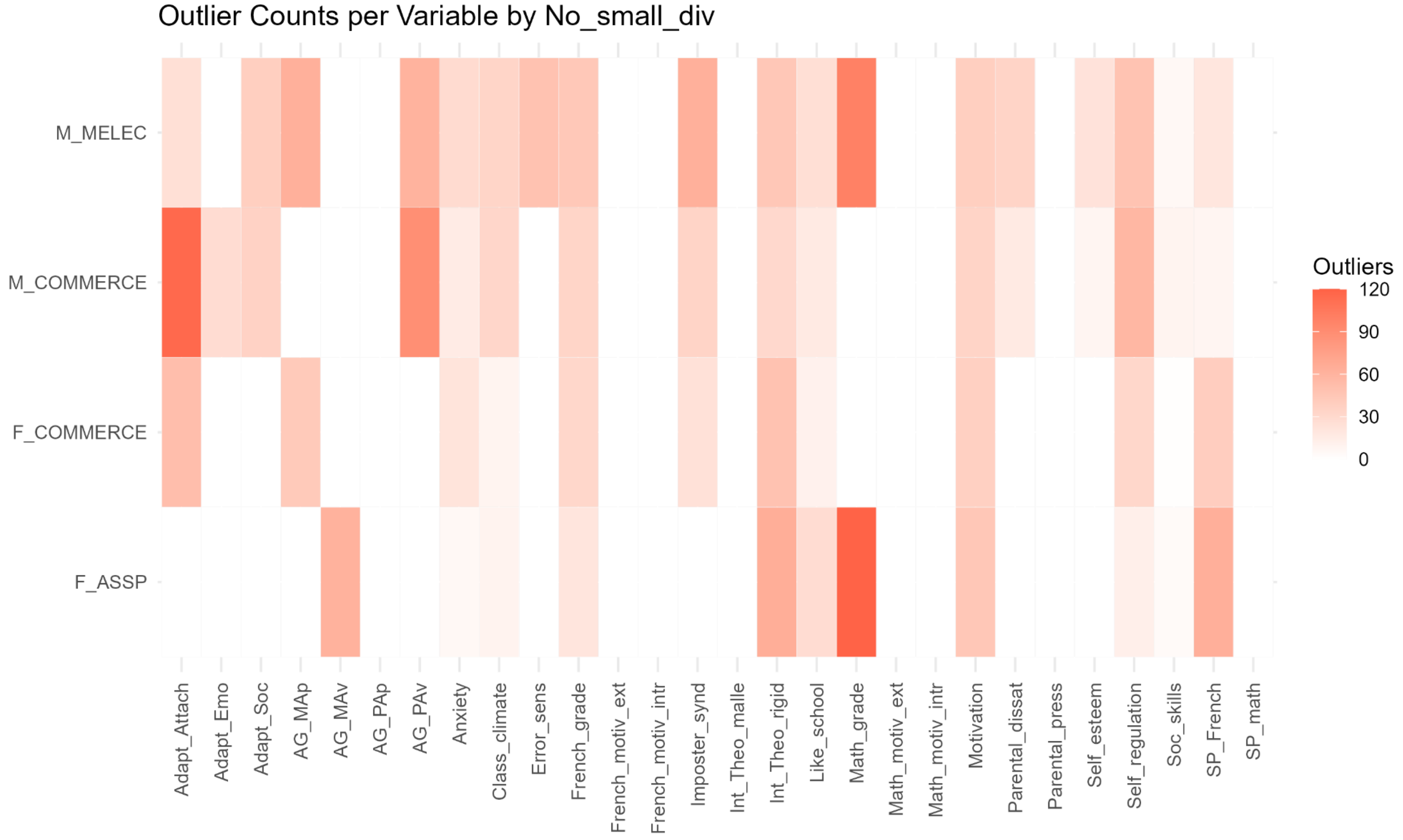

Note: Like_school – To like school or not; Anxiety – School anxiety; Self-regulation – Self-regulation; AG_MAp – Approach Master Goals; AG_MAv – Avoidance Master Goals; AG_PAp – Approach Performance Goals; AG_PAv – Avoidance Performance Goals; Emo_adapt –

Emotional and personal adaptation; Soc_adapt – Social adapting; Attach_adapt – Attachment to the institution; Class_climate – Classroom climate; SP_French and SP_math– Self-perception in Maths and French; Soc_skills – Social skills; Self_esteem – Global self-esteem; Motivation – Motivation for cognitive activities; French_motiv_ext and Math_motiv_ext – External motivation for maths and French; French_motiv_intr and Math_motiv_intr – Intrinsic motivation for maths and french; Parental_dissat – Perception of parental dissatisfaction; Parental_press – Parental pressure; Error_sens – Error sensitivity; Imposter_synd – Feeling like an academic imposter; Int_Theo_rigid – Theory of entity intelligence (rigid); Int_Theo_malle – Theory of incremental intelligence (malleable); French_grade and Math_grade – Maths and French Competencies

Figure S3 Distributions for all study variables divided by track

Figure S3 Univariate normality for study variables in F_ASSP, M_MELEC, M_Commerce and F_commerce

F_ASSP

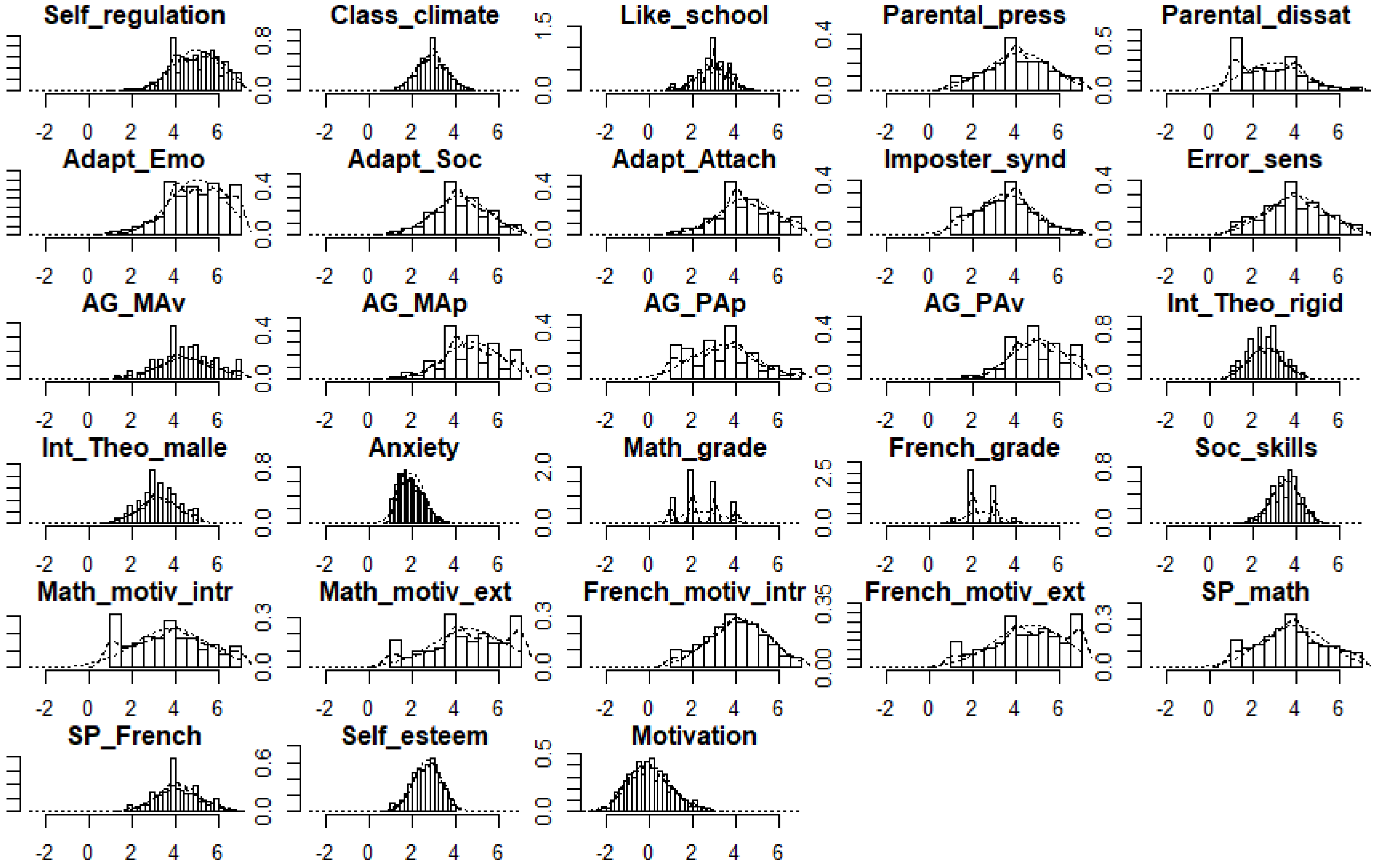
Self_regulation
Class_climate
Like_school
Parental_press
Parental_dissat
Adapt_Emo
Adapt_Soc
Adapt_Attach
Imposter_synd
Error_sens
AG_MAv
AG_MAp
AG_PAp
AG_PAv
Int_Theo_rigid
Int_Theo_malle
Anxiety
Math_grade
French_grade
Soc_skills
Math_motiv_intr
Math_motiv_ext
French_motiv_intr
French_motiv_ext
SP_math
SP_French
Self_esteem
Motivation

M_MELEC

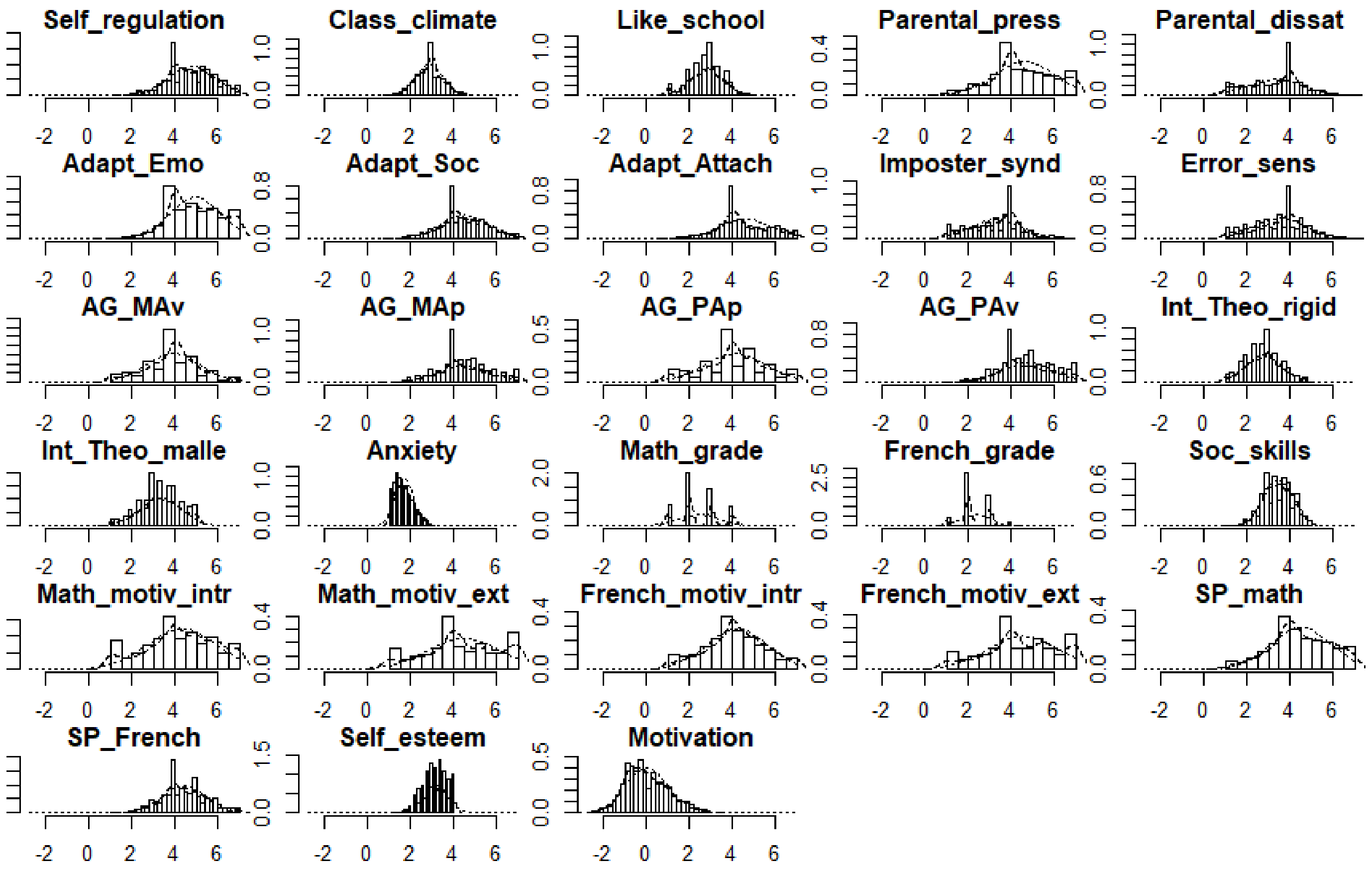
Self_regulation
Class_climate
Like_school
Parental_press
Parental_dissat
Adapt_Emo
Adapt_Soc
Adapt_Attach
Imposter_synd
Error_sens
AG_MAv
AG_MAp
AG_PAp
AG_PAv
Int_Theo_rigid
Int_Theo_malle
Anxiety
Math_grade
French_grade
Soc_skills
Math_motiv_intr
Math_motiv_ext
French_motiv_intr
French_motiv_ext
SP_math
SP_French
Self_esteem
Motivation

M_Commerce

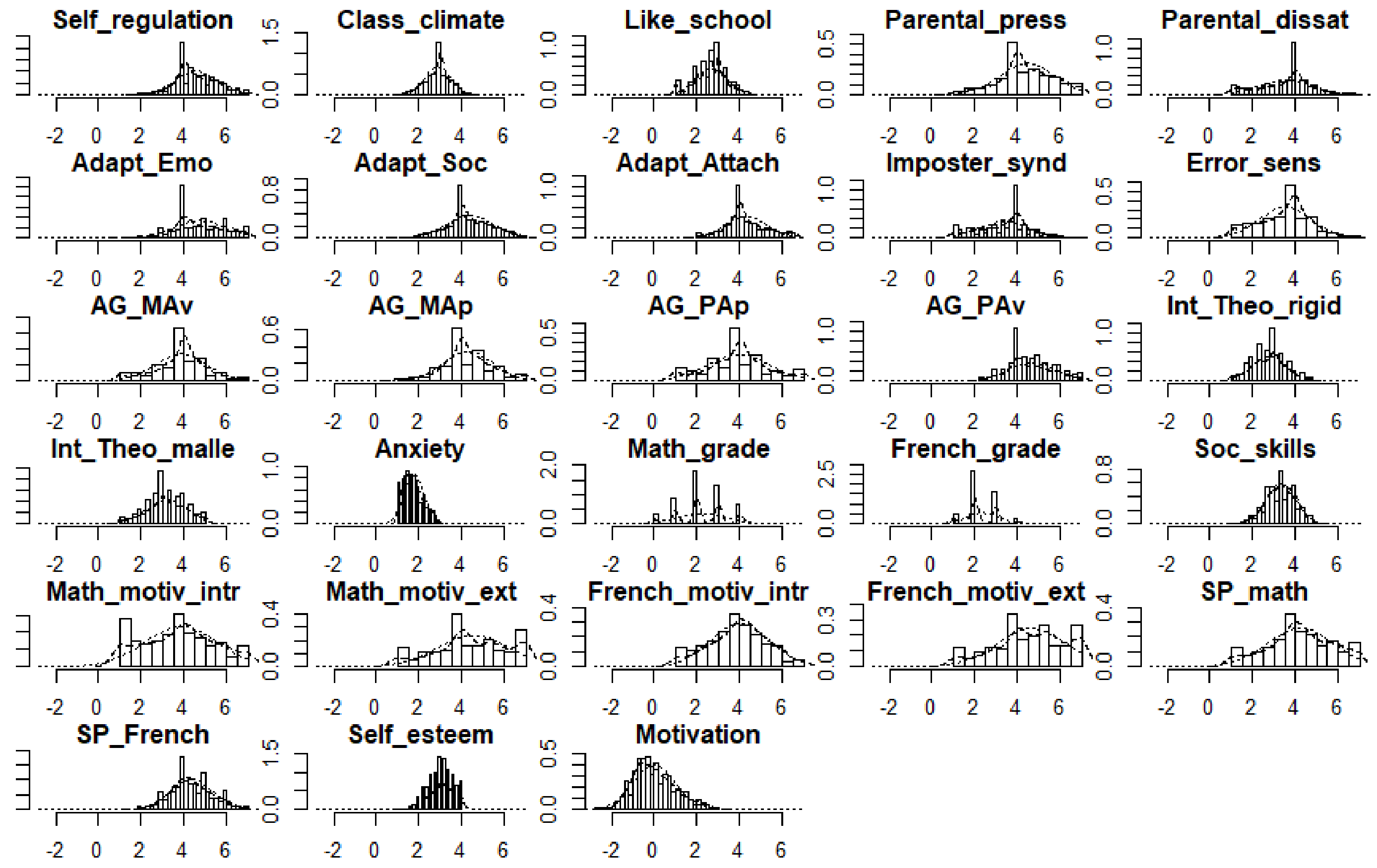
Self_regulation
Class_climate
Like_school
Parental_press
Parental_dissat
Adapt_Emo
Adapt_Soc
Adapt_Attach
Imposter_synd
Error_sens
AG_MAv
AG_MAp
AG_PAp
AG_PAv
Int_Theo_rigid
Int_Theo_malle
Anxiety
Math_grade
French_grade
Soc_skills
Math_motiv_intr
Math_motiv_ext
French_motiv_intr
French_motiv_ext
SP_math
SP_French
Self_esteem
Motivation

F_Commerce

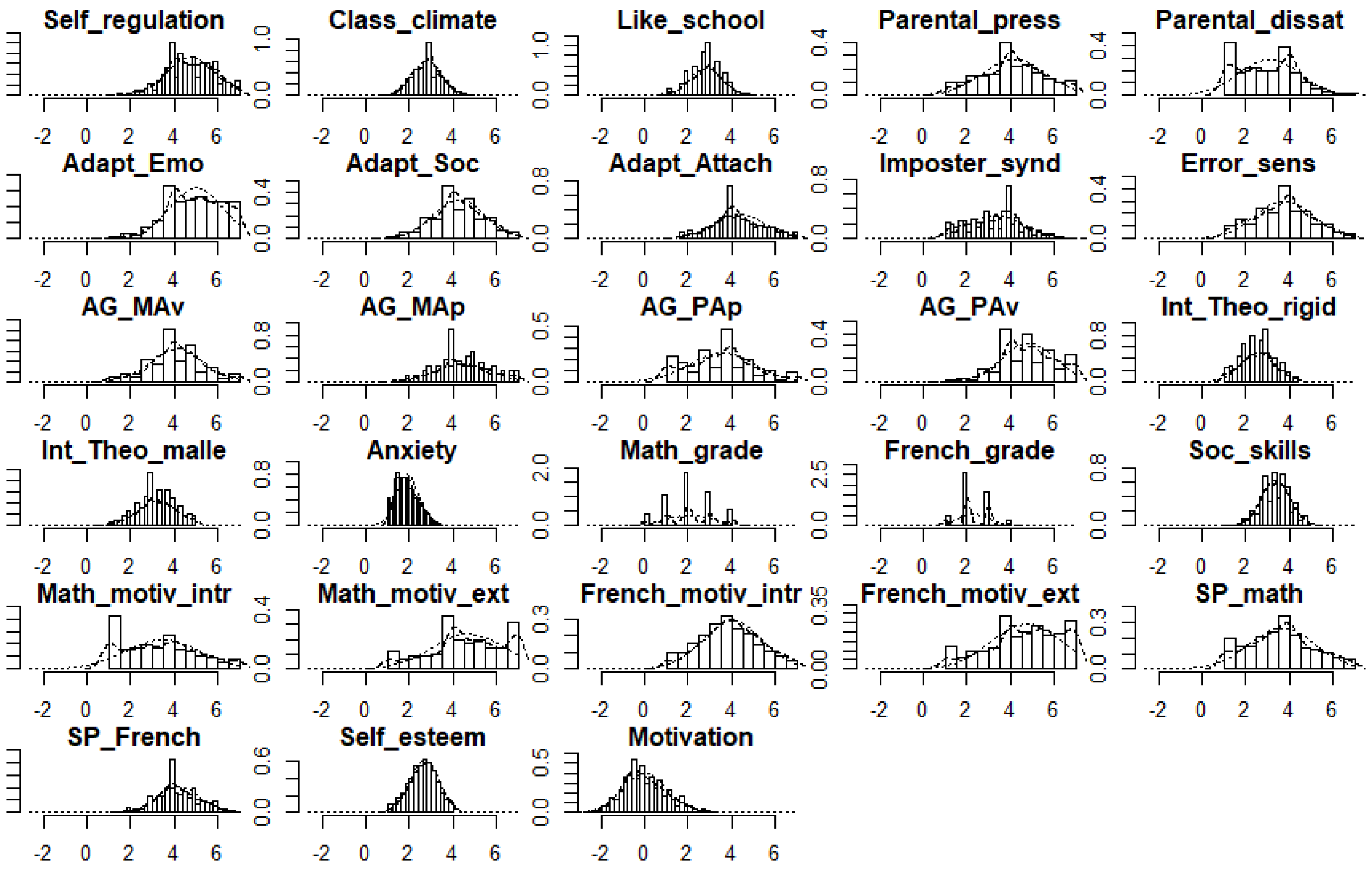

Figure S4 Network plot and centrality indices for Males is ASSP group

Figure S4 Network plot (Panel A) and centrality indices (Panel B) for Males is ASSP group

Panel A

M_ASSP

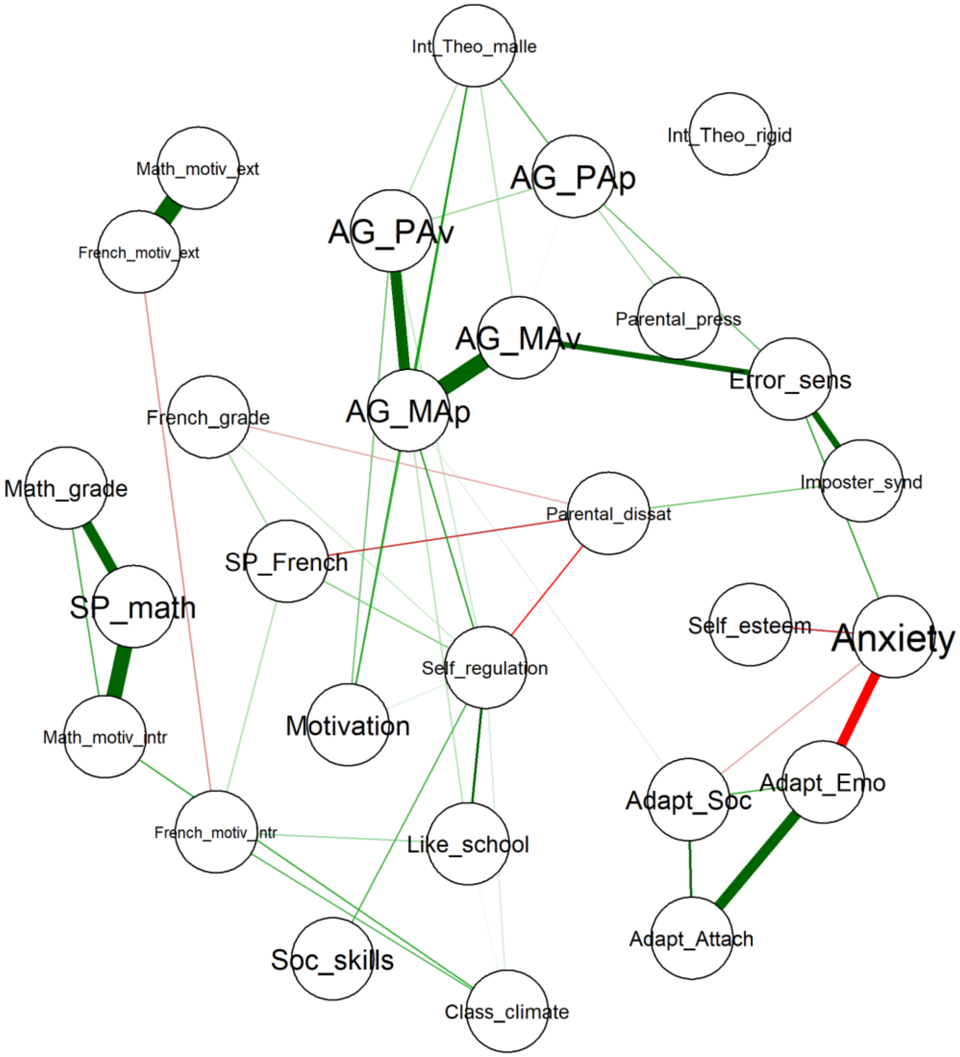
Int_Theo_malle
Int_Theo_rigid
Math_motiv_ext
French_motiv_ext
AG_PAp
AG_PAv
AG_MAv
Parental_press
Error_sens
AG_MAp
French_grade
Math_grade
Imposter_synd
Parental_dissat
SP_French
SP_math
Self_esteem
Anxiety
Self_regulation
Motivation
Math_motiv_intr
Adapt_Emo
Adapt_Soc
French_motiv_intr
Like_school
Adapt_Attach
Soc_skills
Class_climate

Panel B

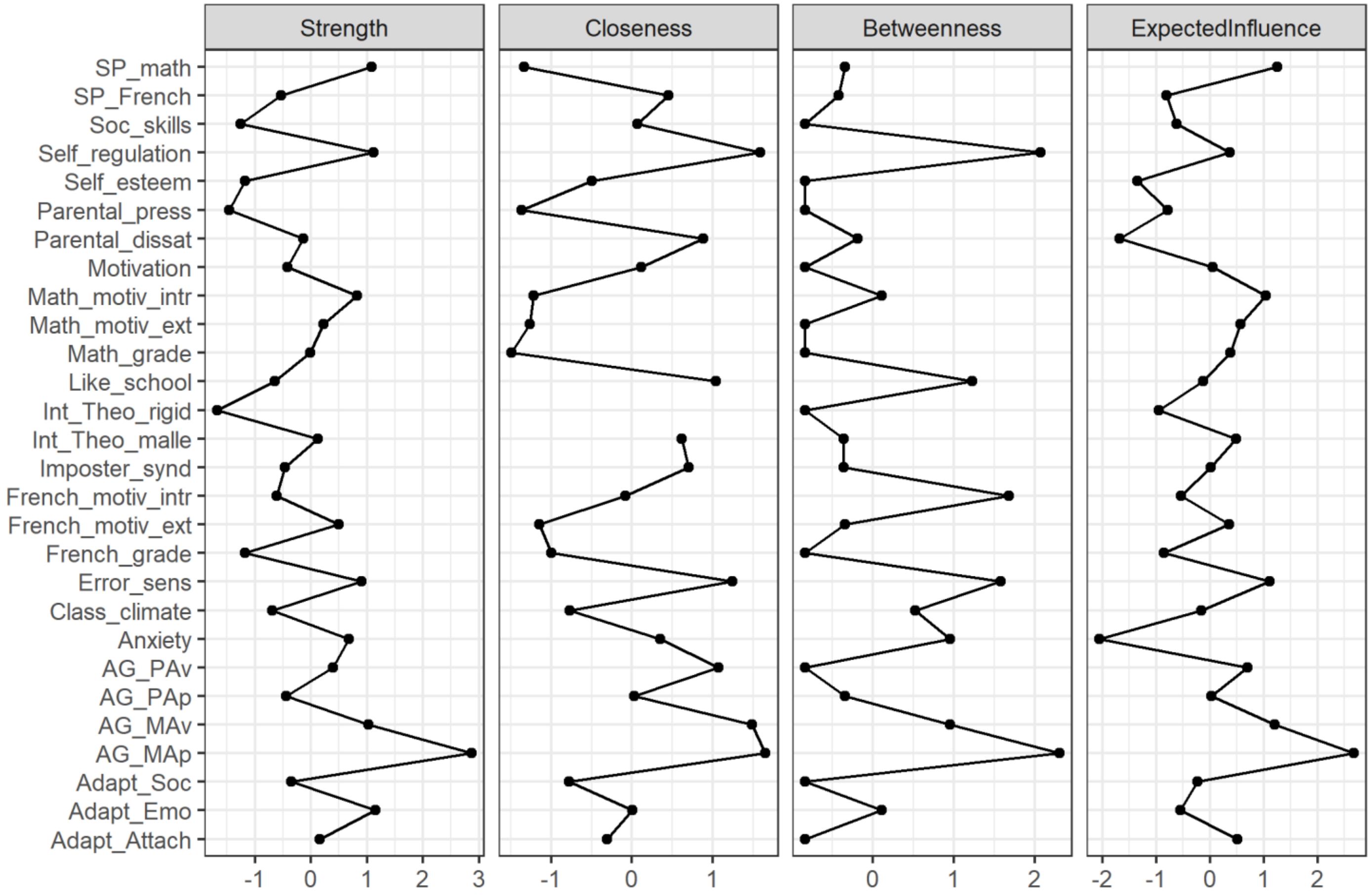

Strength
Closeness
Betweenness
ExpectedInfluence
SP_math
SP_French
Soc_skills
Self_regulation
Self_esteem
Parental_press
Parental_dissat
Motivation
Math_motiv_intr
Math_motiv_ext
Math_grade
Like_school
Int_Theo_rigid
Int_Theo_malle
Imposter_synd
French_motiv_intr
French_motiv_ext
French_grade
Error_sens
Class_climate
Anxiety
AG_PAv
AG_PAp
AG_MAv
AG_MAp
Adapt_Soc
Adapt_Emo
Adapt_Attach
-1 0 1 2 3
-1 0 1
0 1 2
-2 -1 0 1 2

Table S2 Welch ANOVA results for study variables

Table S2 Welch ANOVA results for study variables

Variable	n	F	DFn	DFd	p	Partial η^2
AG_MAp	8065	51.99	3	4119.394	<.001	**0.02**
AG_MAv	8109	119.68	3	4135.382	<.001	**0.042**
AG_PAp	8171	71.32	3	4166.691	<.001	**0.026**
AG_PAv	8021	18.24	3	4090.117	<.001	0.007
Adapt_Attach	7967	17.26	3	4149.728	<.001	0.006
Adapt_Emo	8132	10.89	3	4166.031	<.001	0.004
Adapt_Soc	8085	23.68	3	4126.619	<.001	0.009
Anxiety	8103	139.44	3	4204.489	<.001	**0.047**
Class_climate	8065	12.49	3	4133.266	<.001	0.005
Error_sens	8111	55.21	3	4159.613	<.001	**0.02**
French_grade	8049	9.26	3	4060.88	<.001	0.003
French_motiv_ext	8197	3.37	3	4204.266	0.018	0.001
French_motiv_intr	8197	4.95	3	4188.061	0.002	0.002
Imposter_synd	8038	18.94	3	4140.091	<.001	0.007
Int_Theo_malle	8173	6.26	3	4131.292	<.001	0.002
Int_Theo_rigid	7983	33.47	3	4062.453	<.001	0.013
Like_school	8069	65.18	3	4102.681	<.001	**0.024**
Math_grade	7961	53.06	3	4015.471	<.001	**0.021**
Math_motiv_ext	8200	6.14	3	4196.603	<.001	0.002
Math_motiv_intr	8200	62.14	3	4186.409	<.001	**0.022**
Motivation	7992	72.7	3	4042.521	<.001	**0.029**
Parental_dissat	8108	71.47	3	4169.978	<.001	**0.025**
Parental_press	8160	41.89	3	4176.647	<.001	0.015
SP_French	8065	43.8	3	4137.626	<.001	0.016
SP_math	8200	113.84	3	4192.33	<.001	**0.038**
Self_esteem	8161	298.87	3	4191.524	<.001	**0.096**
Self_regulation	7994	38.88	3	4053.075	<.001	0.014

Soc_skills	8178	23.65	3	4143.13	<.001	0.008

Note: effects larger than small (>.01) as per Cohen's threshold are in bold

Table S3 Posthoc comparisons among 4 groups for all study variables

Table S3 Posthoc comparisons for ANOVA, Bonferronni adjusted

Variable	group1	group2	p	p.adj	p.adj.signif	Cohen's d
AG_MAv	F_ASSP	M_COMMERCE	5.06E-58	3.04E-57	****	0.528944
Anxiety	F_ASSP	M_MELEC	4.46E-69	2.68E-68	****	0.511648
AG_MAv	F_ASSP	M_MELEC	1.23E-50	7.35E-50	****	0.444696
Anxiety	F_COMMERCE	M_MELEC	4.26E-37	2.55E-36	****	0.43788
Like_school	F_ASSP	M_COMMERCE	5.18E-39	3.11E-38	****	0.427538
Anxiety	F_ASSP	M_COMMERCE	1.48E-39	8.89E-39	****	0.405394
AG_MAp	F_ASSP	M_COMMERCE	9.77E-37	5.86E-36	****	0.399931
Self_regulation	F_ASSP	M_COMMERCE	1.91E-24	1.15E-23	****	0.338249
Math_grade	F_ASSP	F_COMMERCE	2.57E-28	1.54E-27	****	0.336406
Error_sens	F_ASSP	M_MELEC	1.22E-29	7.33E-29	****	0.336393
Anxiety	F_COMMERCE	M_COMMERCE	8.87E-20	5.32E-19	****	0.325872
Error_sens	F_ASSP	M_COMMERCE	1.91E-21	1.15E-20	****	0.298274
AG_MAv	F_ASSP	F_COMMERCE	1.37E-20	8.24E-20	****	0.286761
Like_school	F_ASSP	M_MELEC	6.56E-21	3.94E-20	****	0.275194
AG_MAp	F_COMMERCE	M_COMMERCE	1.81E-12	1.08E-11	****	0.24665
Soc_skills	F_ASSP	M_COMMERCE	4.23E-14	2.54E-13	****	0.245072
Like_school	F_COMMERCE	M_COMMERCE	1.29E-11	7.72E-11	****	0.23813
AG_MAv	F_COMMERCE	M_COMMERCE	4.82E-12	2.89E-11	****	0.238017
Math_grade	F_ASSP	M_COMMERCE	7.16E-11	4.29E-10	****	0.211985
Error_sens	F_COMMERCE	M_MELEC	4.97E-10	2.98E-09	****	0.206935
AG_PAv	F_ASSP	M_COMMERCE	5.87E-09	3.52E-08	****	0.196011
Imposter_synd	F_ASSP	M_MELEC	1.54E-11	9.23E-11	****	0.195547
Like_school	F_ASSP	F_COMMERCE	5.34E-10	3.21E-09	****	0.191219

Soc_skills	F_ASSP	F_COMMERCE	1.43E-09	8.58E-09	****	0.187762
Self_regulation	F_ASSP	M_MELEC	2.96E-10	1.77E-09	****	0.185757
Imposter_synd	F_ASSP	M_COMMERCE	9.37E-09	5.62E-08	****	0.178679
Self_regulation	F_COMMERCE	M_COMMERCE	8.80E-07	5.28E-06	****	0.17368
Error_sens	F_COMMERCE	M_COMMERCE	6.47E-07	3.88E-06	****	0.171087
Adapt_Emo	F_ASSP	M_COMMERCE	2.29E-07	1.37E-06	****	0.167736
AG_PAv	F_ASSP	M_MELEC	1.09E-08	6.54E-08	****	0.167693
Math_motiv_intr	F_ASSP	F_COMMERCE	3.84E-08	2.30E-07	****	0.164303
Class_climate	F_ASSP	F_COMMERCE	2.97E-08	1.78E-07	****	0.163561
AG_MAp	F_ASSP	F_COMMERCE	8.69E-08	5.22E-07	****	0.161192
AG_MAv	F_COMMERCE	M_MELEC	5.01E-07	3.01E-06	****	0.158654
Self_regulation	F_ASSP	F_COMMERCE	1.30E-07	7.82E-07	****	0.158445
AG_MAp	F_ASSP	M_MELEC	1.85E-07	1.11E-06	****	0.153488
AG_PAv	F_ASSP	F_COMMERCE	1.38E-07	8.31E-07	****	0.152958
Imposter_synd	F_ASSP	F_COMMERCE	5.42E-07	3.25E-06	****	0.144856
French_grade	F_ASSP	M_MELEC	5.61E-06	3.37E-05	****	0.135572
Class_climate	F_ASSP	M_COMMERCE	3.63E-05	0.000218	***	0.131881
Adapt_Attach	F_ASSP	M_COMMERCE	6.92E-05	0.000415	***	0.1295
Math_motiv_ext	F_COMMERCE	M_MELEC	5.75E-05	0.000345	***	0.129145
Error_sens	F_ASSP	F_COMMERCE	9.39E-06	5.63E-05	****	0.129117
Class_climate	F_ASSP	M_MELEC	1.16E-05	6.95E-05	****	0.127596
French_grade	F_ASSP	F_COMMERCE	6.78E-05	0.000407	***	0.12141
Adapt_Emo	F_COMMERCE	M_COMMERCE	0.000895	0.00537	**	0.118671
Int_Theo_malle	F_ASSP	M_COMMERCE	0.000467	0.0028	**	0.112753
Adapt_Emo	F_ASSP	M_MELEC	0.000106	0.000636	***	0.111524
Soc_skills	F_ASSP	M_MELEC	0.000203	0.00122	**	0.108268
Anxiety	M_COMMERCE	M_MELEC	0.0057	0.0342	*	0.105868
French_grade	F_ASSP	M_COMMERCE	0.00168	0.0101	*	0.102576
Motivation	F_ASSP	F_COMMERCE	0.00162	0.00974	**	0.10177
French_motiv_ext	F_COMMERCE	M_MELEC	0.00177	0.0106	*	0.100892
Adapt_Attach	F_ASSP	F_COMMERCE	0.00047	0.00282	**	0.100395
Parental_dissat	M_COMMERCE	M_MELEC	0.0058	0.0348	*	0.100055

Anxiety	F_ASSP	F_COMMERCE	0.000364	0.00219	**	0.099892
French_motiv_intr	F_ASSP	M_COMMERCE	0.00331	0.0198	*	0.09385
Math_motiv_ext	F_ASSP	M_MELEC	0.00179	0.0107	*	0.090381
Like_school	F_COMMERCE	M_MELEC	0.00566	0.034	*	0.088411
SP_math	F_ASSP	F_COMMERCE	0.00442	0.0265	*	0.083875
Math_motiv_ext	F_COMMERCE	M_COMMERCE	0.0194	0.116	ns	0.081808
French_motiv_intr	F_ASSP	F_COMMERCE	0.0137	0.082	ns	0.074832
Adapt_Emo	F_COMMERCE	M_MELEC	0.0529	0.317	ns	0.061864
Int_Theo_malle	F_COMMERCE	M_COMMERCE	0.0769	0.461	ns	0.061368
Soc_skills	F_COMMERCE	M_COMMERCE	0.0788	0.473	ns	0.061088
French_motiv_ext	F_ASSP	M_MELEC	0.0436	0.261	ns	0.058407
French_motiv_ext	M_COMMERCE	M_MELEC	0.114	0.683	ns	0.053829
Int_Theo_malle	F_ASSP	F_COMMERCE	0.09	0.54	ns	0.052869
Adapt_Emo	F_ASSP	F_COMMERCE	0.088	0.528	ns	0.050689
Imposter_synd	F_COMMERCE	M_MELEC	0.138	0.827	ns	0.050453
Math_motiv_ext	M_COMMERCE	M_MELEC	0.157	0.94	ns	0.047602
French_motiv_ext	F_COMMERCE	M_COMMERCE	0.179	1	ns	0.04705
Int_Theo_rigid	F_ASSP	F_COMMERCE	0.129	0.775	ns	0.046843
Math_motiv_ext	F_ASSP	M_COMMERCE	0.177	1	ns	0.043001
Imposter_synd	F_COMMERCE	M_COMMERCE	0.321	1	ns	0.035718
AG_PAv	F_COMMERCE	M_COMMERCE	0.357	1	ns	0.03275
French_grade	M_COMMERCE	M_MELEC	0.35	1	ns	0.031699
Error_sens	M_COMMERCE	M_MELEC	0.411	1	ns	0.029357
Adapt_Attach	F_COMMERCE	M_COMMERCE	0.479	1	ns	0.028049
Self_regulation	F_COMMERCE	M_MELEC	0.418	1	ns	0.025876
Self_esteem	F_ASSP	F_COMMERCE	0.411	1	ns	0.023646
French_motiv_intr	F_COMMERCE	M_COMMERCE	0.562	1	ns	0.020319
Adapt_Soc	F_ASSP	F_COMMERCE	0.603	1	ns	0.015338
Imposter_synd	M_COMMERCE	M_MELEC	0.698	1	ns	0.014226
French_grade	F_COMMERCE	M_MELEC	0.678	1	ns	0.013302
AG_PAv	F_COMMERCE	M_MELEC	0.744	1	ns	0.010225
Parental_press	F_ASSP	F_COMMERCE	0.806	1	ns	0.007291

French_motiv_ext	F_ASSP	M_COMMERCE	0.87	1	ns	0.005191
Class_climate	M_COMMERCE	M_MELEC	0.892	1	ns	-0.00481
French_motiv_intr	F_ASSP	M_MELEC	0.833	1	ns	-0.00612
AG_MAp	F_COMMERCE	M_MELEC	0.807	1	ns	-0.00811
Math_grade	F_ASSP	M_MELEC	0.773	1	ns	-0.00936
Int_Theo_rigid	M_COMMERCE	M_MELEC	0.723	1	ns	-0.01191
French_grade	F_COMMERCE	M_COMMERCE	0.594	1	ns	-0.0183
AG_PAv	M_COMMERCE	M_MELEC	0.528	1	ns	-0.02307
Int_Theo_malle	F_ASSP	M_MELEC	0.337	1	ns	-0.02805
Class_climate	F_COMMERCE	M_COMMERCE	0.342	1	ns	-0.03375
Math_motiv_ext	F_ASSP	F_COMMERCE	0.207	1	ns	-0.03804
Class_climate	F_COMMERCE	M_MELEC	0.24	1	ns	-0.03847
French_motiv_ext	F_ASSP	F_COMMERCE	0.168	1	ns	-0.04118
AG_PAp	F_ASSP	F_COMMERCE	0.16	0.961	ns	-0.04233
SP_French	F_ASSP	F_COMMERCE	0.131	0.788	ns	-0.04667
Math_motiv_intr	F_ASSP	M_COMMERCE	0.0886	0.532	ns	-0.05382
Adapt_Emo	M_COMMERCE	M_MELEC	0.116	0.696	ns	-0.05446
Adapt_Attach	F_ASSP	M_MELEC	0.0282	0.169	ns	-0.06063
SP_French	M_COMMERCE	M_MELEC	0.035	0.21	ns	-0.06989
Soc_skills	F_COMMERCE	M_MELEC	0.023	0.138	ns	-0.072
AG_MAv	M_COMMERCE	M_MELEC	0.0206	0.124	ns	-0.07674
Int_Theo_malle	F_COMMERCE	M_MELEC	0.0139	0.0836	ns	-0.07841
French_motiv_intr	F_COMMERCE	M_MELEC	0.0125	0.0753	ns	-0.08
Adapt_Soc	M_COMMERCE	M_MELEC	0.00682	0.0409	*	-0.09524
French_motiv_intr	M_COMMERCE	M_MELEC	0.0032	0.0192	*	-0.09831
AG_PAp	M_COMMERCE	M_MELEC	0.00256	0.0154	*	-0.10185
Parental_dissat	F_ASSP	F_COMMERCE	0.000175	0.00105	**	-0.10831
Self_esteem	M_COMMERCE	M_MELEC	0.00179	0.0107	*	-0.11281
Math_grade	F_COMMERCE	M_COMMERCE	0.00034	0.00204	**	-0.1131
Adapt_Soc	F_ASSP	M_COMMERCE	0.000267	0.0016	**	-0.11732
Soc_skills	M_COMMERCE	M_MELEC	8.41E-05	0.000505	***	-0.12829
Int_Theo_malle	M_COMMERCE	M_MELEC	3.78E-05	0.000227	***	-0.13367

Adapt_Soc	F_COMMERCE	M_COMMERCE	0.000134	0.000807	***	-0.13424
Parental_press	F_ASSP	M_COMMERCE	2.39E-05	0.000144	***	-0.13743
SP_math	M_COMMERCE	M_MELEC	4.26E-05	0.000256	***	-0.14188
Parental_press	F_COMMERCE	M_COMMERCE	3.87E-05	0.000232	***	-0.14327
Like_school	M_COMMERCE	M_MELEC	1.74E-05	0.000105	***	-0.14342
Self_regulation	M_COMMERCE	M_MELEC	2.15E-05	0.000129	***	-0.14864
Motivation	F_ASSP	M_COMMERCE	6.55E-06	3.93E-05	****	-0.14924
Parental_press	M_COMMERCE	M_MELEC	3.23E-06	1.94E-05	****	-0.16024
Adapt_Attach	F_COMMERCE	M_MELEC	1.57E-07	9.45E-07	****	-0.17246
SP_French	F_COMMERCE	M_COMMERCE	9.41E-08	5.64E-07	****	-0.18971
Motivation	M_COMMERCE	M_MELEC	4.47E-10	2.68E-09	****	-0.19983
Int_Theo_rigid	F_ASSP	M_COMMERCE	8.15E-10	4.89E-09	****	-0.20033
Parental_dissat	F_COMMERCE	M_MELEC	4.52E-10	2.71E-09	****	-0.20415
Math_motiv_intr	M_COMMERCE	M_MELEC	1.34E-09	8.02E-09	****	-0.20648
Adapt_Soc	F_ASSP	M_MELEC	7.39E-13	4.44E-12	****	-0.21018
Int_Theo_rigid	F_ASSP	M_MELEC	8.81E-13	5.29E-12	****	-0.21096
Adapt_Attach	M_COMMERCE	M_MELEC	2.30E-08	1.38E-07	****	-0.21189
Math_motiv_intr	F_COMMERCE	M_COMMERCE	2.48E-10	1.49E-09	****	-0.21726
Math_grade	M_COMMERCE	M_MELEC	1.87E-10	1.12E-09	****	-0.21785
Adapt_Soc	F_COMMERCE	M_MELEC	2.24E-12	1.34E-11	****	-0.22779
SP_French	F_ASSP	M_COMMERCE	6.26E-13	3.76E-12	****	-0.23346
AG_PAp	F_COMMERCE	M_COMMERCE	1.46E-11	8.78E-11	****	-0.23466
Int_Theo_rigid	F_COMMERCE	M_COMMERCE	2.97E-12	1.78E-11	****	-0.24594
Motivation	F_COMMERCE	M_COMMERCE	6.25E-12	3.75E-11	****	-0.25264
AG_MAp	M_COMMERCE	M_MELEC	1.30E-13	7.79E-13	****	-0.255
SP_French	F_COMMERCE	M_MELEC	1.63E-15	9.81E-15	****	-0.25549
Int_Theo_rigid	F_COMMERCE	M_MELEC	2.45E-15	1.47E-14	****	-0.25585
Math_motiv_intr	F_ASSP	M_MELEC	5.43E-19	3.26E-18	****	-0.26179
SP_math	F_ASSP	M_COMMERCE	1.57E-17	9.44E-17	****	-0.26582
AG_PAp	F_ASSP	M_COMMERCE	7.29E-18	4.37E-17	****	-0.27636
Parental_press	F_ASSP	M_MELEC	1.05E-23	6.30E-23	****	-0.29355
Parental_dissat	F_COMMERCE	M_COMMERCE	2.34E-17	1.40E-16	****	-0.29641

Parental_press	F_COMMERCE	M_MELEC	7.29E-21	4.37E-20	****	-0.29703
SP_French	F_ASSP	M_MELEC	8.10E-25	4.86E-24	****	-0.29924
Parental_dissat	F_ASSP	M_MELEC	1.79E-26	1.08E-25	****	-0.31318
AG_PAp	F_COMMERCE	M_MELEC	1.08E-25	6.49E-25	****	-0.33733
Math_grade	F_COMMERCE	M_MELEC	5.17E-26	3.10E-25	****	-0.34098
Motivation	F_ASSP	M_MELEC	3.74E-34	2.25E-33	****	-0.35114
SP_math	F_COMMERCE	M_COMMERCE	5.69E-25	3.41E-24	****	-0.353
AG_PAp	F_ASSP	M_MELEC	3.36E-38	2.02E-37	****	-0.37893
Parental_dissat	F_ASSP	M_COMMERCE	1.56E-36	9.37E-36	****	-0.40296
SP_math	F_ASSP	M_MELEC	9.82E-45	5.89E-44	****	-0.41587
Math_motiv_intr	F_COMMERCE	M_MELEC	8.98E-40	5.39E-39	****	-0.42999
Motivation	F_COMMERCE	M_MELEC	1.71E-44	1.02E-43	****	-0.44711
SP_math	F_COMMERCE	M_MELEC	1.55E-53	9.31E-53	****	-0.51206
Self_esteem	F_ASSP	M_COMMERCE	9.69E-73	5.81E-72	****	-0.57818
Self_esteem	F_COMMERCE	M_COMMERCE	1.10E-67	6.58E-67	****	-0.60007
Self_esteem	F_ASSP	M_MELEC	6.29E-119	3.78E-118	****	-0.69658
Self_esteem	F_COMMERCE	M_MELEC	1.88E-106	1.13E-105	****	-0.72192

Table S4 Network edges for 4 groups

Table S4 GGM network edges for 4 groups

F_ASSP

	Self_regulation	Class_climate	Like_school	Parental_press	Parental_dissat	Adapt_Emo	Adapt_Soc	Adapt_Attach	Imposter_synd	Error_sens	AG_MAv	AG_MAp	AG_PAp	AG_PAv	Int_Theo_rigid	Int_Theo_malle	Anxiety	Math_grade	French_grade	Soc_skills	Math_motiv_intr	Math_motiv_ext	French_motiv_intr	French_motiv_ext	SP_math	SP_French	Self_esteem
Self_regulation	1.00																										
Class_climate	0.37	1.00																									

Like_school	0.45	0.36	1.00														
Parental_press	0.10	0.06	0.09	1.00													
Parental_dissat	-0.42	-0.25	-0.26	0.28	1.00												
Adapt_Emo	0.20	0.22	0.16	-0.08	-0.39	1.00											
Adapt_Soc	0.27	0.36	0.28	0.12	-0.18	0.41	1.00										
Adapt_Attach	0.33	0.37	0.38	-0.03	-0.36	0.52	0.47	1.00									
Imposter_synd	-0.06	-0.09	0.02	0.22	0.34	-0.40	-0.04	-0.21	1.00								
Error_sens	0.20	0.03	0.19	0.26	0.13	-0.30	-0.01	-0.12	0.57	1.00							
AG_MAv	0.29	0.12	0.17	0.25	-0.02	-0.08	0.08	0.00	0.28	0.43	1.00						
AG_MAp	0.54	0.28	0.37	0.24	-0.25	0.09	0.21	0.21	0.10	0.34	0.60	1.00					
AG_PAp	0.18	0.07	0.18	0.29	-0.01	-0.11	0.10	0.06	0.19	0.38	0.28	0.30	1.00				
AG_PAv	0.43	0.21	0.26	0.23	-0.23	0.11	0.17	0.17	0.10	0.31	0.52	0.63	0.28	1.00			
Int_Theo_rigid	-0.10	-0.09	-0.08	0.10	0.20	-0.16	-0.02	-0.12	0.21	0.15	0.11	0.00	0.18	0.01	1.00		
Int_Theo_malle	0.19	0.10	0.16	0.24	0.02	-0.01	0.10	0.06	0.13	0.22	0.28	0.34	0.31	0.31	0.18	1.00	
Anxiety	-0.09	-0.20	-0.07	0.03	0.20	-0.46	-0.38	-0.36	0.36	0.38	0.21	0.06	0.08	0.06	0.11	0.06	1.00

Math_grade	0.24	0.12	0.16	0.00	-0.26	0.06	0.10	0.16	0.00	0.04	0.03	0.13	0.18	0.11	-0.03	0.07	-0.07	1.00									
French_grade	0.25	0.12	0.16	0.00	-0.26	0.04	0.04	0.10	-0.03	0.09	0.08	0.21	0.16	0.16	-0.10	0.03	-0.04	0.31	1.00								
Soc_skills	0.41	0.28	0.28	0.04	-0.19	0.11	0.18	0.21	0.05	0.15	0.20	0.32	0.06	0.27	-0.06	0.12	-0.03	0.11	0.14	1.00							
Math_motiv_intr	0.27	0.21	0.31	0.07	-0.14	0.04	0.17	0.20	0.06	0.13	0.06	0.17	0.17	0.12	0.05	0.12	-0.05	0.42	0.01	0.15	1.00						
Math_motiv_ext	0.00	-0.04	-0.08	0.10	0.06	-0.08	0.01	-0.09	0.12	0.12	0.12	0.05	0.12	0.06	0.08	0.09	0.10	-0.07	-0.02	0.00	-0.04	1.00					
French_motiv_intr	0.31	0.31	0.37	0.09	-0.08	0.05	0.19	0.17	0.04	0.17	0.19	0.32	0.13	0.22	0.01	0.12	-0.01	0.04	0.16	0.28	0.28	0.03	1.00				
French_motiv_ext	-0.06	-0.10	-0.15	0.07	0.05	-0.07	-0.02	-0.10	0.07	0.03	0.02	-0.03	0.06	0.02	0.12	0.06	0.06	-0.03	-0.08	-0.06	0.01	0.71	-0.11	1.00			
SP_math	0.20	0.14	0.18	0.02	-0.18	0.10	0.12	0.21	-0.06	-0.05	-0.09	0.04	0.17	0.01	-0.05	0.06	-0.16	0.57	0.06	0.04	0.61	-0.13	-0.01	-0.01	1.00		
SP_French	0.25	0.15	0.21	0.03	-0.18	0.11	0.10	0.14	-0.15	-0.03	-0.02	0.15	0.12	0.11	-0.14	0.01	-0.14	0.04	0.42	0.14	-0.05	-0.06	0.30	-0.13	0.04	1.00	
Self_esteem	0.18	0.21	0.17	-0.03	-0.23	0.28	0.31	0.29	-0.33	-0.27	-0.11	0.02	0.02	0.01	-0.08	-0.01	-0.44	0.06	0.08	0.06	0.11	-0.09	0.11	-0.07	0.17	0.20	1.00
Motivation	0.39	0.24	0.31	0.21	-0.13	0.05	0.27	0.16	0.05	0.17	0.29	0.41	0.24	0.34	0.00	0.22	-0.06	0.13	0.16	0.34	0.24	0.08	0.31	0.03	0.20	0.26	0.14

F_COMM

	Self_regulation	Class_climate	Like_school	Parental_press	Parental_dissat	Adapt_Emo	Adapt_Soc	Adapt_Attach	Imposter_synd	Error_sens	AG_MAv	AG_MAp	AG_PAp	AG_PAv	Int_Theo_rigid	Int_Theo_malle	Anxiety	Math_grade	French_grade	Soc_skills	Math_motiv_intr	Math_motiv_ext	French_motiv_intr	French_motiv_ext	SP_math	SP_French	Self_esteem
Self_regulation	1.00																										

Class_climate	0.38	1.00														
Like_school	0.44	0.40	1.00													
Parental_press	0.18	0.13	0.13	1.00												
Parental_dissat	-0.39	-0.25	-0.22	0.20	1.00											
Adapt_Emo	0.20	0.16	0.16	-0.03	-0.39	1.00										
Adapt_Soc	0.25	0.34	0.29	0.18	-0.18	0.36	1.00									
Adapt_Attach	0.29	0.36	0.32	-0.02	-0.38	0.49	0.42	1.00								
Imposter_synd	-0.06	0.01	0.01	0.19	0.35	-0.42	0.02	-0.21	1.00							
Error_sens	0.20	0.14	0.18	0.26	0.11	-0.30	0.05	-0.08	0.57	1.00						
AG_MAv	0.25	0.15	0.20	0.26	0.00	-0.08	0.10	-0.03	0.26	0.44	1.00					
AG_MAp	0.53	0.34	0.37	0.29	-0.19	0.11	0.20	0.17	0.06	0.33	0.54	1.00				
AG_PAp	0.26	0.16	0.21	0.31	-0.02	-0.12	0.08	0.07	0.21	0.36	0.33	0.33	1.00			
AG_PAv	0.47	0.24	0.25	0.28	-0.23	0.16	0.19	0.14	0.05	0.30	0.46	0.60	0.31	1.00		
Int_Theo_rigid	-0.10	-0.09	-0.08	0.12	0.22	-0.20	-0.04	-0.15	0.25	0.20	0.11	0.00	0.17	-0.02	1.00	
Int_Theo_malle	0.25	0.17	0.16	0.23	-0.02	-0.01	0.12	0.07	0.13	0.24	0.31	0.36	0.33	0.29	0.19	1.00

Anxiety	-0.12	-0.10	-0.09	0.00	0.15	-0.41	-0.32	-0.25	0.32	0.34	0.18	0.02	0.10	0.02	0.18	0.06	1.00										
Math_grade	0.21	0.14	0.18	0.03	-0.24	0.08	0.07	0.17	0.02	0.06	0.05	0.10	0.14	0.13	-0.04	0.10	-0.03	1.00									
French_grade	0.26	0.14	0.18	0.03	-0.26	0.02	0.05	0.12	-0.04	0.11	0.06	0.15	0.17	0.17	-0.09	0.06	-0.01	0.32	1.00								
Soc_skills	0.42	0.26	0.28	0.05	-0.24	0.14	0.18	0.19	0.01	0.10	0.15	0.31	0.05	0.27	-0.05	0.14	-0.02	0.12	0.14	1.00							
Math_motiv_intr	0.26	0.27	0.31	0.08	-0.15	0.04	0.14	0.21	0.06	0.10	0.09	0.21	0.14	0.14	0.01	0.16	-0.06	0.44	0.01	0.13	1.00						
Math_motiv_ext	0.03	0.00	-0.08	0.11	0.03	-0.07	-0.01	-0.05	0.08	0.08	0.13	0.03	0.09	0.15	0.08	0.10	0.09	-0.07	0.01	0.03	-0.01	1.00					
French_motiv_intr	0.38	0.34	0.41	0.07	-0.18	0.10	0.23	0.20	0.01	0.15	0.15	0.33	0.12	0.25	-0.03	0.16	-0.06	0.08	0.22	0.28	0.26	0.01	1.00				
French_motiv_ext	-0.01	-0.07	-0.14	0.13	0.09	-0.08	-0.02	-0.08	0.13	0.07	0.06	-0.03	0.07	0.09	0.09	0.07	0.09	-0.04	-0.05	-0.04	-0.01	0.69	-0.11	1.00			
SP_math	0.19	0.18	0.19	0.01	-0.18	0.09	0.09	0.20	-0.04	-0.02	-0.03	0.10	0.11	0.06	-0.03	0.06	-0.10	0.57	0.05	0.07	0.64	-0.12	0.06	-0.05	1.00		
SP_French	0.30	0.12	0.23	0.04	-0.17	0.12	0.05	0.14	-0.14	-0.01	0.00	0.16	0.13	0.16	-0.13	0.02	-0.13	0.04	0.38	0.11	-0.05	-0.07	0.34	-0.16	0.02	1.00	
Self_esteem	0.18	0.20	0.18	0.04	-0.18	0.25	0.29	0.29	-0.27	-0.22	-0.13	0.07	0.05	0.04	-0.11	0.02	-0.40	0.08	0.02	0.09	0.13	-0.03	0.12	-0.06	0.15	0.16	1.00
Motivation	0.43	0.29	0.29	0.19	-0.16	0.07	0.26	0.12	0.04	0.20	0.27	0.42	0.24	0.37	-0.02	0.22	-0.04	0.14	0.15	0.32	0.22	0.11	0.33	0.04	0.17	0.22	0.15

M_COMM

	Self_regulation	Class_climate	Like_school	Parental_press	Parental_dissat	Adapt_Emo	Adapt_Soc	Adapt_Attach	Imposter_synd	Error_sens	AG_MAv	AG_MAp	AG_PAp	AG_PAv	Int_Theo_rigid	Int_Theo_male	Anxiety	Math_grade	French_grade	Soc_skills	Math_motiv_intr	Math_motiv_ext	French_motiv_intr	French_motiv_ext	SP_math	SP_French	Self_esteem
Self_reg	1.00																										

ulation																
Class_climate	0.35	1.00														
Like_school	0.35	0.37	1.00													
Parental_press	0.17	0.11	0.09	1.00												
Parental_dissat	-0.31	-0.14	-0.15	0.18	1.00											
Adapt_Emo	0.13	0.07	0.02	0.00	-0.35	1.00										
Adapt_Soc	0.27	0.28	0.23	0.24	-0.07	0.30	1.00									
Adapt_Attach	0.25	0.25	0.25	0.00	-0.33	0.46	0.34	1.00								
Imposter_synd	-0.11	0.03	0.03	0.09	0.39	-0.56	-0.05	-0.27	1.00							
Error_sens	0.10	0.13	0.24	0.22	0.22	-0.42	0.03	-0.15	0.58	1.00						
AG_MAv	0.19	0.12	0.19	0.19	0.13	-0.18	0.07	-0.05	0.27	0.42	1.00					
AG_MAp	0.44	0.26	0.32	0.26	-0.15	0.02	0.21	0.13	0.06	0.30	0.49	1.00				
AG_PAp	0.22	0.14	0.18	0.29	-0.01	-0.06	0.17	0.04	0.12	0.35	0.28	0.37	1.00			
AG_PAv	0.37	0.15	0.14	0.34	-0.14	0.13	0.22	0.14	-0.08	0.16	0.37	0.48	0.35	1.00		
Int_Theo_rigid	-0.10	-0.05	-0.09	0.06	0.21	-0.14	-0.02	-0.15	0.25	0.18	0.13	-0.01	0.11	-0.02	1.00	
Int_The	0.24	0.15	0.19	0.16	-0.04	0.05	0.14	0.11	0.08	0.19	0.23	0.31	0.23	0.26	0.02	1.00

o_malle																											
Anxiety	-0.17	-0.09	-0.01	-0.05	-0.11	-0.30	-0.32	-0.22	0.21	0.21	0.19	-0.01	-0.04	-0.04	0.07	-0.05	1.00										
Math_grade	0.16	0.08	0.11	0.06	-0.15	0.01	0.07	0.08	0.00	0.04	0.02	0.08	0.17	0.04	-0.04	0.02	-0.05	1.00									
French_grade	0.19	0.11	0.09	0.09	-0.16	0.03	0.09	0.03	-0.06	0.03	0.00	0.13	0.16	0.12	-0.08	0.01	-0.06	0.30	1.00								
Soc_skills	0.29	0.24	0.21	0.08	-0.13	0.08	0.18	0.17	-0.04	0.09	0.17	0.26	0.07	0.22	-0.07	0.11	-0.04	0.10	0.12	1.00							
Math_motiv_intr	0.24	0.24	0.33	0.05	-0.13	0.02	0.15	0.21	0.02	0.14	0.10	0.20	0.13	0.07	-0.04	0.10	-0.04	0.44	0.00	0.18	1.00						
Math_motiv_ext	0.02	-0.01	-0.09	0.09	0.05	-0.08	0.02	-0.11	0.11	0.10	0.12	0.07	0.13	0.13	0.16	0.07	0.04	-0.07	-0.03	-0.01	-0.11	1.00					
French_motiv_intr	0.30	0.29	0.40	0.13	-0.11	0.07	0.18	0.18	-0.02	0.11	0.14	0.26	0.13	0.15	-0.07	0.10	-0.07	-0.01	0.15	0.24	0.29	-0.01	1.00				
French_motiv_ext	-0.03	-0.05	-0.14	0.06	0.04	-0.02	0.02	-0.08	0.07	0.06	0.08	0.01	0.09	0.10	0.17	0.04	0.08	-0.04	-0.03	-0.05	-0.06	0.67	-0.04	1.00			
SP_math	0.18	0.10	0.12	0.01	-0.16	0.05	0.10	0.13	-0.08	-0.02	-0.09	0.06	0.12	0.02	-0.01	0.02	-0.11	0.62	0.04	0.10	0.61	-0.15	0.02	-0.06	1.00		
SP_French	0.22	0.07	0.15	0.05	-0.16	0.07	0.14	0.10	-0.14	-0.02	-0.04	0.15	0.15	0.13	-0.08	0.04	-0.15	0.07	0.40	0.12	0.00	-0.05	0.29	-0.06	0.05	1.00	
Self_esteem	0.14	0.08	0.09	0.01	-0.19	0.22	0.29	0.18	-0.21	-0.16	-0.10	0.10	0.11	0.04	-0.09	0.04	-0.37	0.09	0.10	0.05	0.10	-0.03	0.11	-0.03	0.11	0.21	1.00
Motivation	0.38	0.23	0.23	0.23	-0.16	0.11	0.29	0.17	-0.05	0.12	0.17	0.36	0.31	0.32	-0.02	0.13	-0.11	0.20	0.19	0.31	0.29	0.07	0.27	0.03	0.26	0.24	0.13
M_MELEC																											
	Self_regulation	Class_climate	Like_school	Parental_press	Parental_dissat	Adapt_Emo	Adapt_Soc	Adapt_Attach	Imposter_synd	Error_sens	AG_MAv	AG_MAp	AG_PAp	AG_PAv	Int_Theo_rigid	Int_Theo_malle	Anxiety	Math_grade	French_grade	Soc_skills	Math_motiv_intr	Math_motiv_ext	French_motiv_intr	French_motiv_ext	SP_math	SP_French	Self_esteem

Self_regulation	1.00														
Class_climate	0.37	1.00													
Like_school	0.42	0.40	1.00												
Parental_press	0.23	0.08	0.12	1.00											
Parental_dissat	-0.35	-0.20	-0.15	0.13	1.00										
Adapt_Emo	0.18	0.08	0.09	0.05	-0.42	1.00									
Adapt_Soc	0.30	0.31	0.25	0.16	-0.18	0.37	1.00								
Adapt_Attach	0.33	0.31	0.32	0.04	-0.42	0.54	0.43	1.00							
Imposter_synd	-0.12	-0.03	0.05	0.04	0.43	-0.51	-0.13	-0.31	1.00						
Error_sens	0.16	0.11	0.21	0.21	0.27	-0.36	0.00	-0.15	0.55	1.00					
AG_MAv	0.21	0.13	0.20	0.21	0.12	-0.16	0.07	-0.06	0.30	0.46	1.00				
AG_MAp	0.54	0.27	0.36	0.31	-0.17	0.11	0.25	0.21	-0.02	0.29	0.51	1.00			
AG_PAp	0.29	0.16	0.18	0.35	-0.02	-0.03	0.15	0.05	0.11	0.35	0.39	0.44	1.00		
AG_PAv	0.42	0.17	0.20	0.30	-0.14	0.14	0.25	0.21	-0.02	0.23	0.39	0.55	0.36	1.00	
Int_Theo_rigid	-0.10	-0.08	-0.07	0.08	0.19	-0.19	-0.07	-0.13	0.22	0.19	0.16	-0.01	0.11	0.01	1.00

Int_Theo_malle	0.29	0.22	0.27	0.22	-0.06	0.07	0.18	0.20	0.06	0.24	0.28	0.40	0.32	0.32	0.05	1.00											
Anxiety	-0.11	-0.10	-0.01	-0.03	0.19	-0.37	-0.28	-0.28	0.29	0.31	0.19	-0.01	0.01	0.01	0.11	0.02	1.00										
Math_grade	0.24	0.17	0.10	0.04	-0.24	0.05	0.04	0.14	-0.07	0.01	0.00	0.13	0.14	0.05	-0.02	0.06	-0.05	1.00									
French_grade	0.27	0.16	0.11	0.07	-0.29	0.09	0.17	0.15	-0.09	0.01	0.07	0.20	0.17	0.17	-0.05	0.05	-0.05	0.32	1.00								
Soc_skills	0.39	0.29	0.24	0.11	-0.20	0.13	0.25	0.19	-0.05	0.09	0.12	0.31	0.12	0.25	-0.10	0.19	-0.06	0.15	0.17	1.00							
Math_motiv_intr	0.41	0.30	0.38	0.11	-0.16	0.06	0.21	0.24	0.00	0.15	0.11	0.31	0.22	0.21	-0.03	0.24	-0.04	0.38	0.06	0.26	1.00						
Math_motiv_ext	-0.05	-0.08	-0.12	0.10	0.12	-0.09	0.01	-0.08	0.14	0.12	0.13	0.03	0.09	0.13	0.09	0.06	0.10	-0.10	-0.01	-0.02	-0.06	1.00					
French_motiv_intr	0.36	0.31	0.39	0.15	-0.14	0.07	0.20	0.21	-0.04	0.13	0.13	0.31	0.18	0.26	-0.05	0.23	-0.05	0.02	0.18	0.27	0.43	-0.01	1.00				
French_motiv_ext	-0.08	-0.08	-0.18	0.09	0.08	-0.08	-0.02	-0.08	0.10	0.07	0.07	-0.02	0.05	0.07	0.08	0.02	0.07	-0.02	-0.03	-0.09	-0.02	0.72	-0.12	1.00			
SP_math	0.29	0.17	0.15	0.07	-0.23	0.09	0.10	0.19	-0.12	-0.03	-0.09	0.16	0.15	0.05	-0.07	0.04	-0.14	0.62	0.10	0.14	0.55	-0.16	0.10	-0.05	1.00		
SP_French	0.29	0.13	0.17	0.09	-0.26	0.17	0.19	0.18	-0.18	-0.06	-0.03	0.19	0.12	0.12	-0.11	0.01	-0.19	0.07	0.43	0.16	0.04	-0.05	0.31	-0.13	0.14	1.00	
Self_esteem	0.21	0.18	0.07	0.06	-0.25	0.25	0.25	0.24	-0.25	-0.15	-0.10	0.12	0.08	0.07	-0.09	0.04	-0.36	0.13	0.15	0.11	0.16	-0.04	0.15	-0.03	0.17	0.23	1.00
Motivation	0.48	0.25	0.27	0.24	-0.21	0.14	0.30	0.20	-0.07	0.11	0.21	0.44	0.30	0.36	-0.05	0.26	-0.09	0.17	0.22	0.40	0.33	0.03	0.30	0.00	0.25	0.27	0.18

Node: Like_school – To like school or not; Anxiety – School anxiety; Self-regulation – Self-regulation; AG_MAp – Approach Master Goals; AG_MAv – Avoidance Master Goals; AG_PAp – Approach Performance Goals; AG_PAv – Avoidance Performance Goals; Emo_adapt – Emotional and personal adaptation; Soc_adapt – Social adapting; Attach_adapt – Attachment to the institution; Class_climate – Classroom

climate; SP_French and SP_math– Self-perception in Maths and French; Soc_skills – Social skills; Self_esteem – Global self-esteem; Motivation – Motivation for cognitive activities; French_motiv_ext and Math_motiv_ext – External motivation for maths and French; French_motiv_intr and Math_motiv_intr – Intrinsic motivation for maths and french; Parental_dissat – Perception of parental dissatisfaction; Parental_press – Parental pressure; Error_sens – Error sensitivity; Imposter_synd – Feeling like an academic imposter; Int_Theo_rigid – Theory of entity intelligence (rigid); Int_Theo_malle – Theory of incremental intelligence (malleable); French_grade and Math_grade – Maths and French Competencies

Figure S5 Cluster division for the four groups

Figure S5 Network clusters for 4 groups

Panel A: M_MELEC	Panel B: F_ASSP

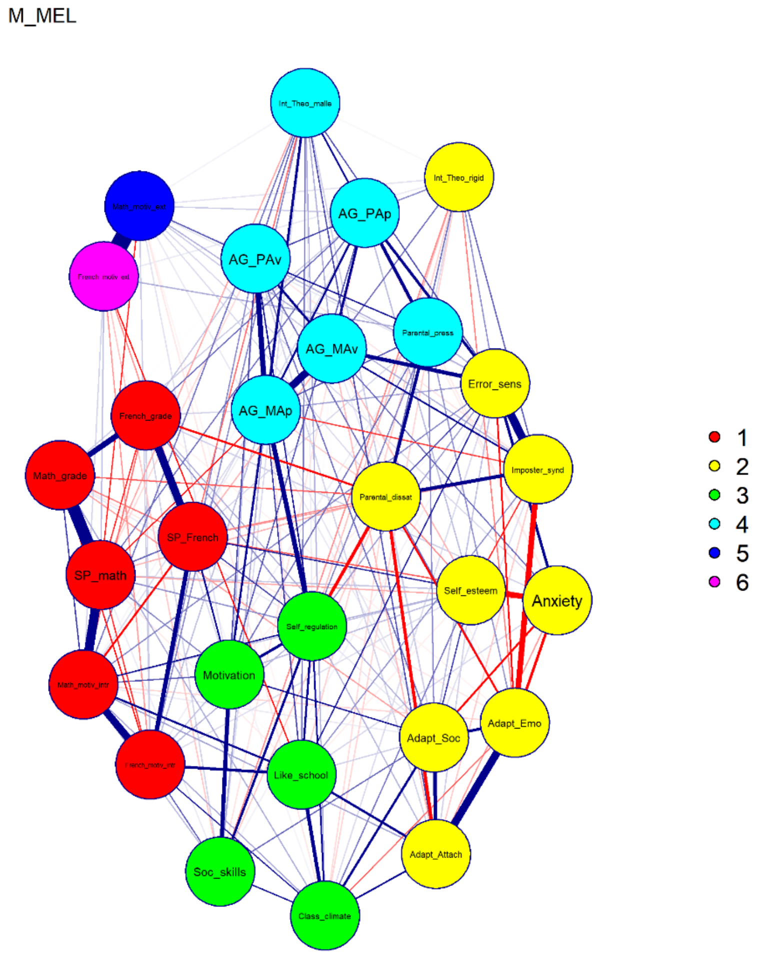

Panel C: M_Commerce

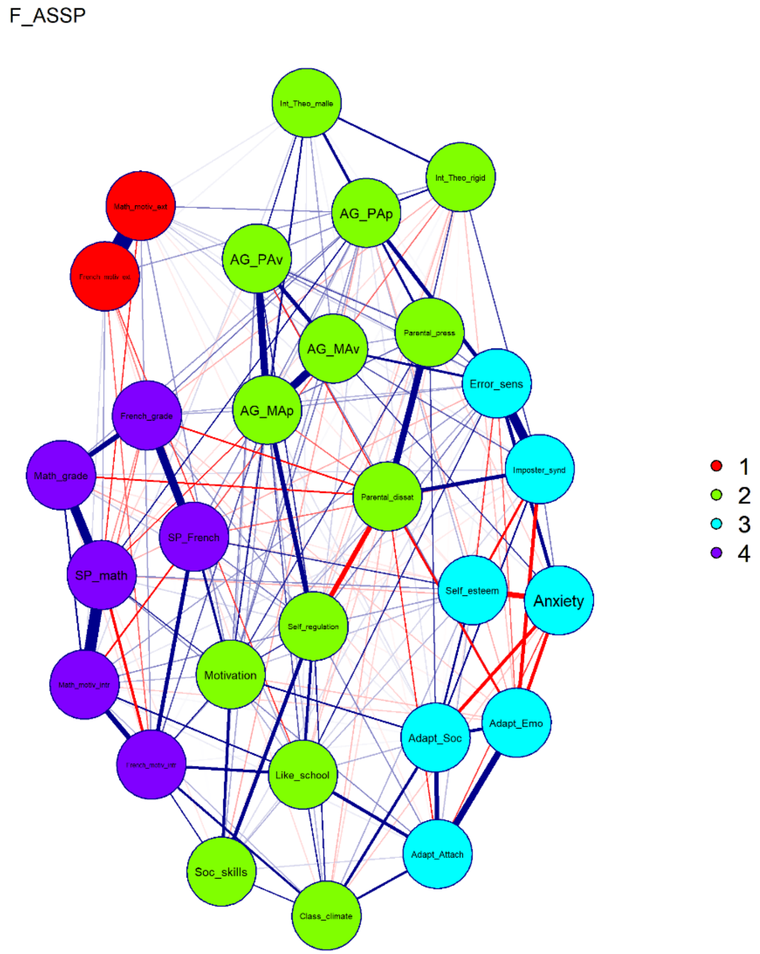

Panel D: F_Commerce

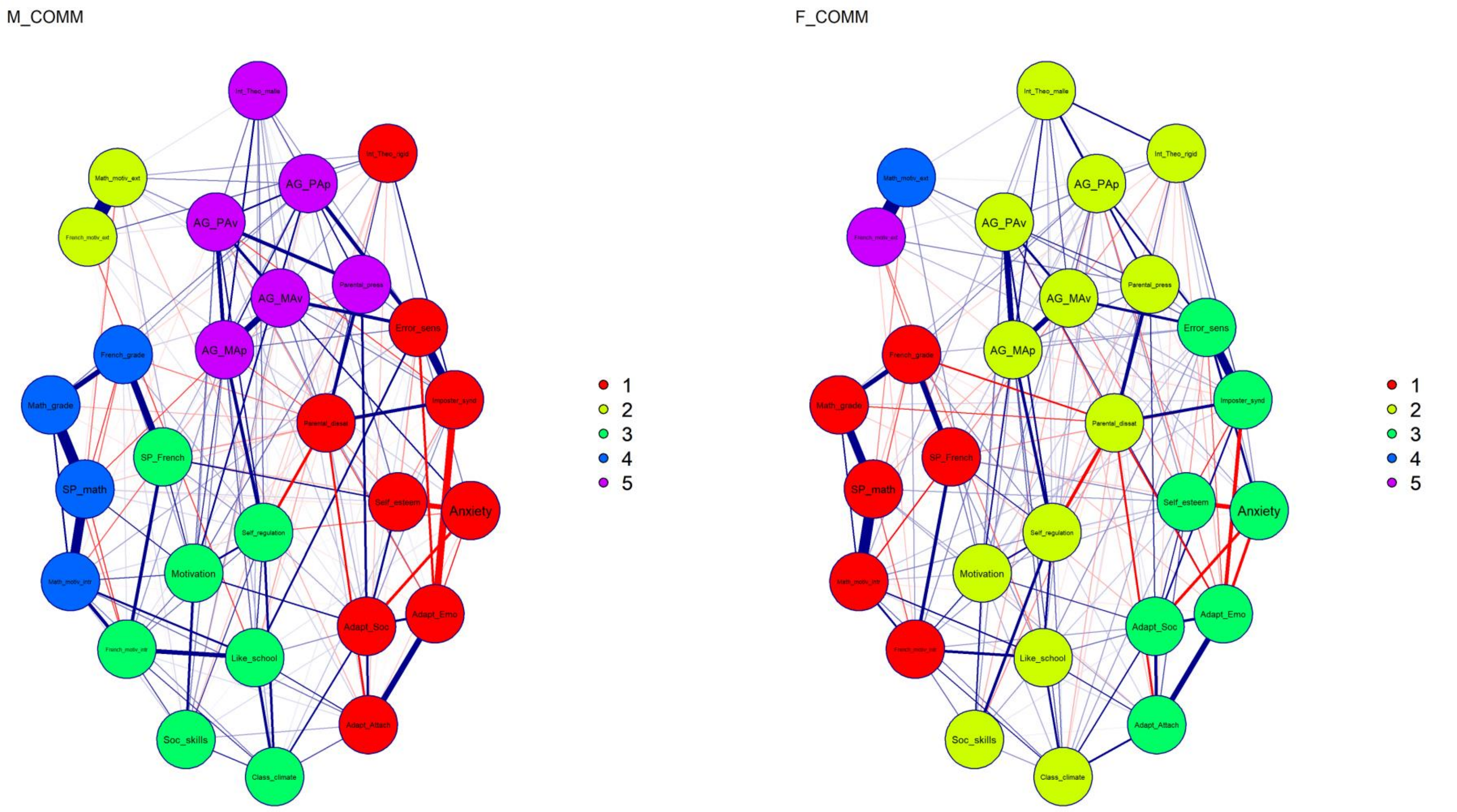

Note: Blue lines indicate positive connections; red lines indicate negative connections; the thickness of the lines represent the strength of the connection; clusters are color-coded, with different clusters marked with different colors. Clusters were inferred using *iqgraph* package for R (Csárdi et al., 2025); Like_school – To like school or not; Anxiety – School anxiety; Self-regulation – Self-regulation; AG_MAp – Approach Master Goals; AG_MAv – Avoidance Master Goals; AG_PAp – Approach Performance Goals; AG_PAv – Avoidance Performance Goals; Emo_adapt – Emotional and personal adaptation; Soc_adapt – Social adapting; Attach_adapt – Attachment to the institution; Class_climate – Classroom climate; SP_French and

SP_math– Self-perception in Maths and French; Soc_skills – Social skills; Self_esteem – Global self-esteem; Motivation – Motivation for cognitive activities; French_motiv_ext and Math_motiv_ext – External motivation for maths and French; French_motiv_intr and Math_motiv_intr – Intrinsic motivation for maths and french; Parental_dissat – Perception of parental dissatisfaction; Parental_press – Parental pressure; Error_sens – Error sensitivity; Imposter_synd – Feeling like an academic imposter; Int_Theo_rigid – Theory of entity intelligence (rigid); Int_Theo_malle – Theory of incremental intelligence (malleable); French_grade and Math_grade – Maths and French Competencies

Figure S6 Bridge centrality for cluster analysis

Figure S6 Bridge centrality indices for network analysis in 4 groups

Panel A: M_MELEC	Panel B: F_ASSP

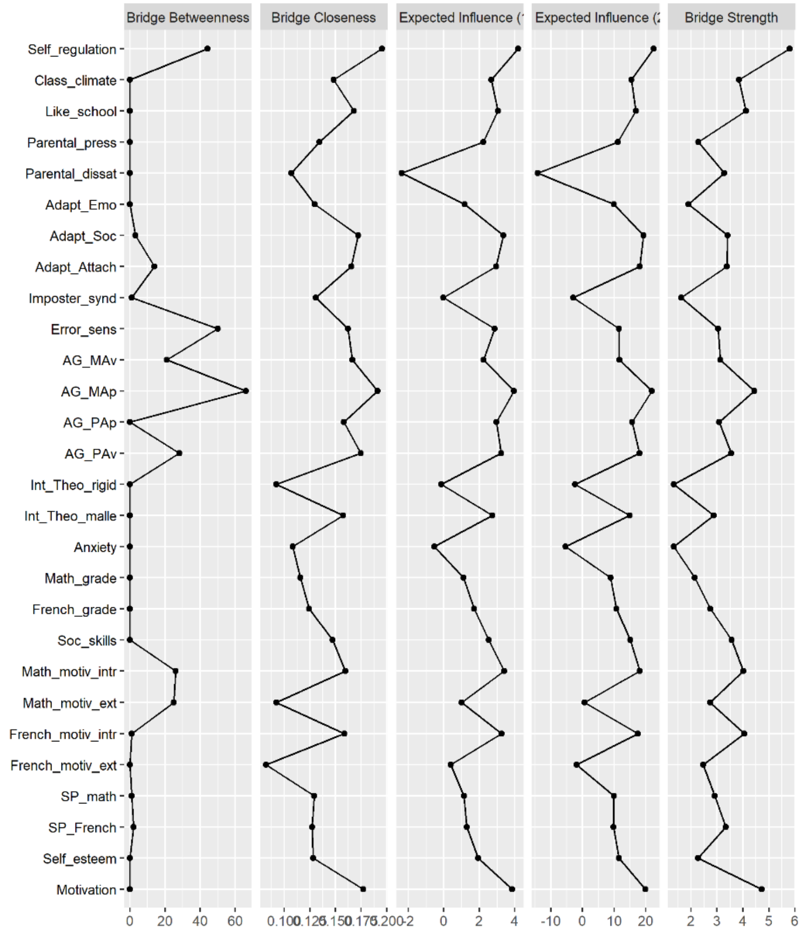

Panel C: M_Commerce

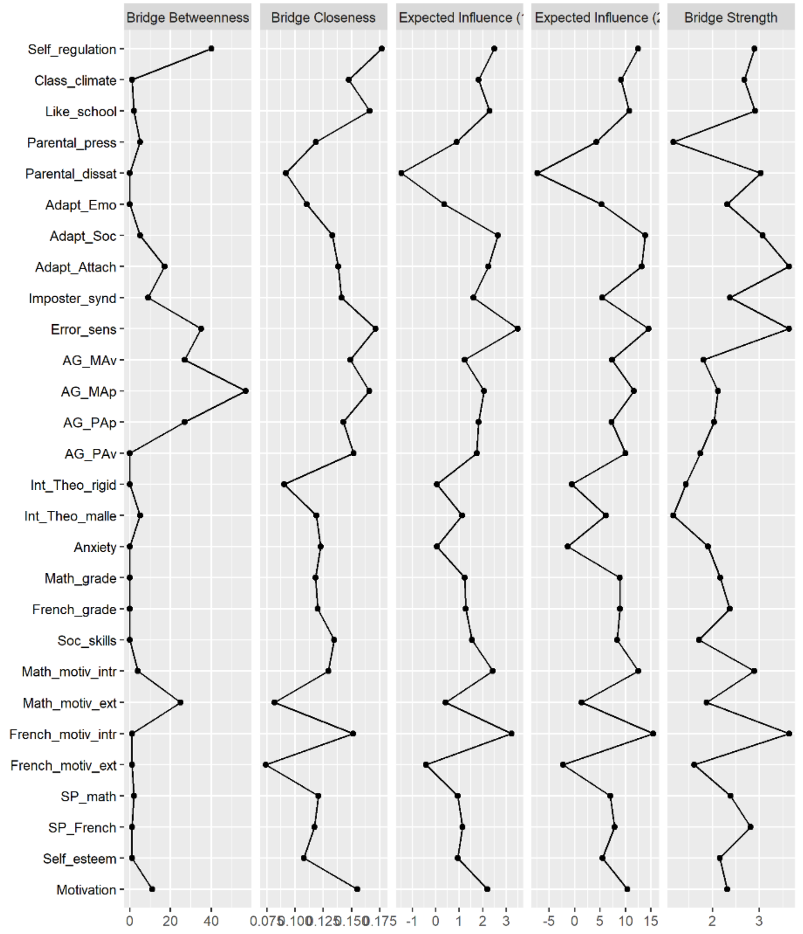

Panel D: F_Commerce

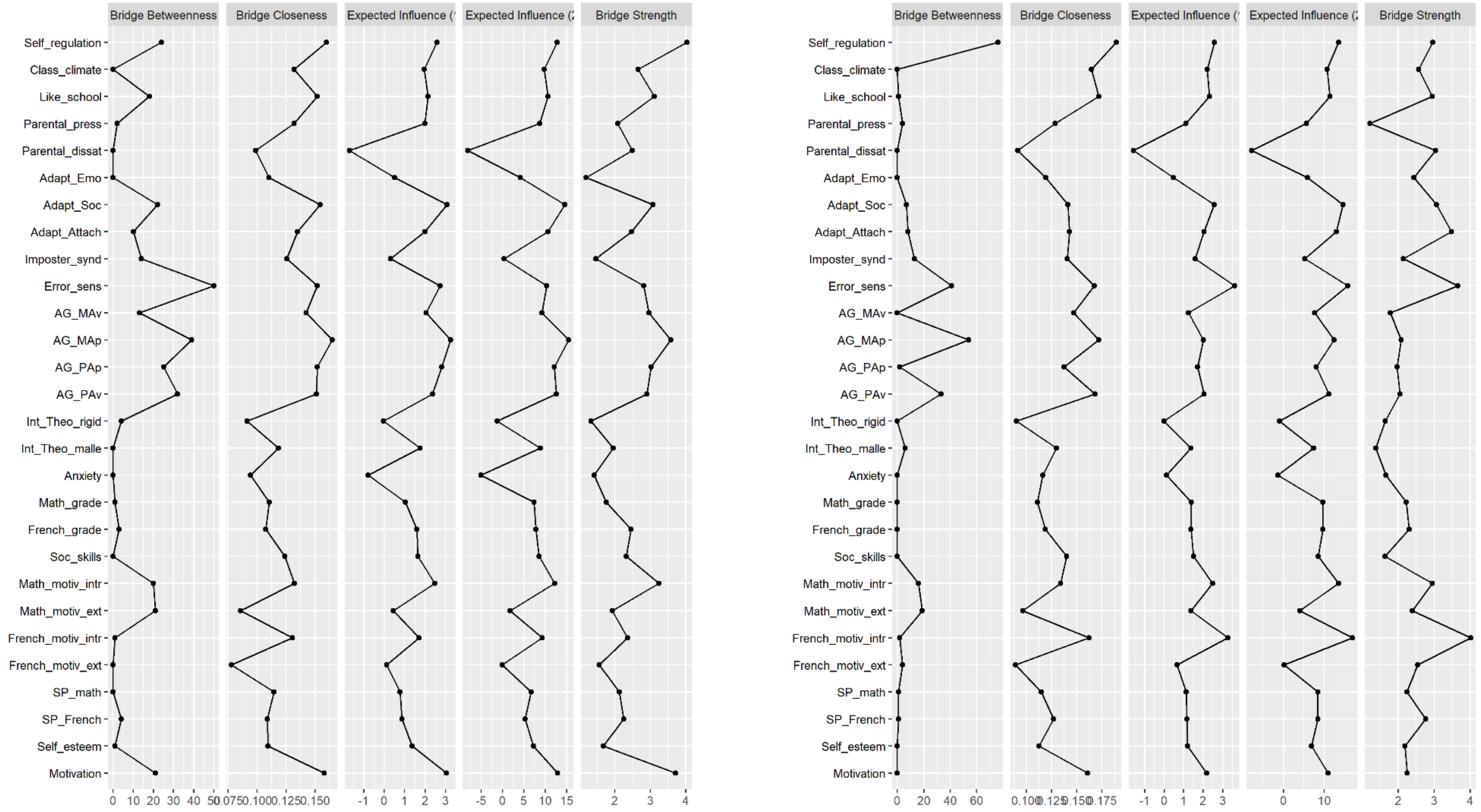

Note: Like_school – To like school or not; Anxiety – School anxiety; Self-regulation – Self-regulation; AG_MAp – Approach Master Goals; AG_MAv – Avoidance Master Goals; AG_PAp – Approach Performance Goals; AG_PAv – Avoidance Performance Goals; Emo_adapt – Emotional and personal adaptation; Soc_adapt – Social adapting; Attach_adapt – Attachment to the institution; Class_climate – Classroom climate; SP_French and SP_math– Self-perception in Maths and French; Soc_skills – Social skills; Self_esteem – Global self-esteem; Motivation – Motivation for cognitive activities; French_motiv_ext and Math_motiv_ext – External motivation for maths and French; French_motiv_intr and Math_motiv_intr – Intrinsic motivation for maths and french; Parental_dissat – Perception of parental dissatisfaction; Parental_press – Parental pressure; Error_sens – Error sensitivity; Imposter_synd – Feeling like an academic imposter; Int_Theo_rigid – Theory of entity intelligence (rigid); Int_Theo_malle – Theory of incremental intelligence (malleable); French_grade and Math_grade – Maths and French Competencies

Figure S7 Full lust of centrality indices for EBIC-glasso network

Figure S7 Centrality indices for network analysis in 4 groups

Panel A: M_MELEC

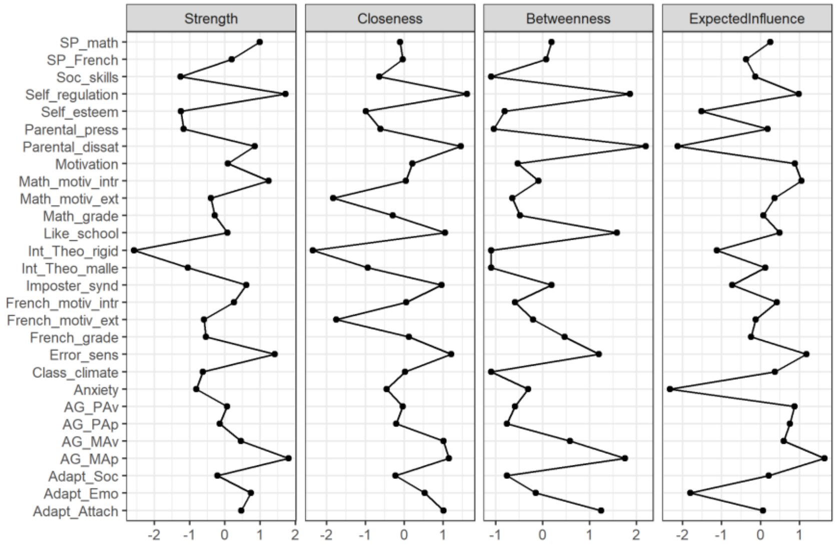

Panel B: F_ASSP

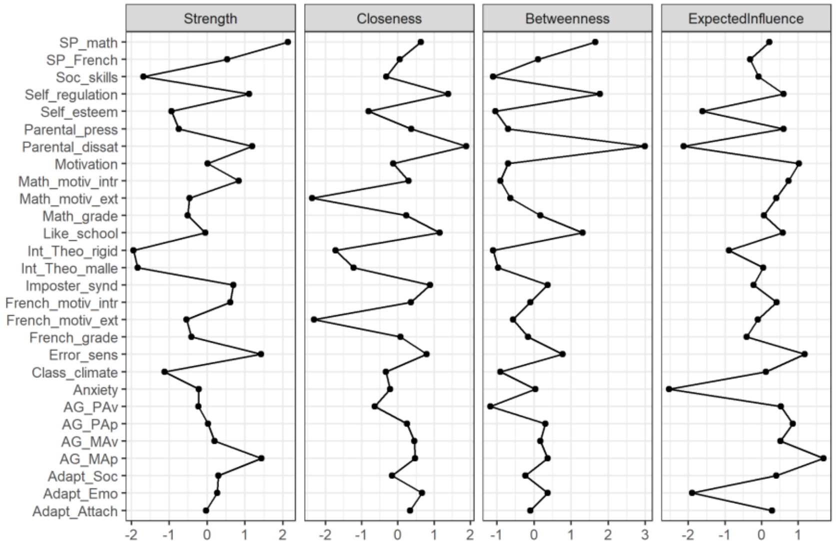

Panel C: M_Commerce

Panel D: F_Commerce

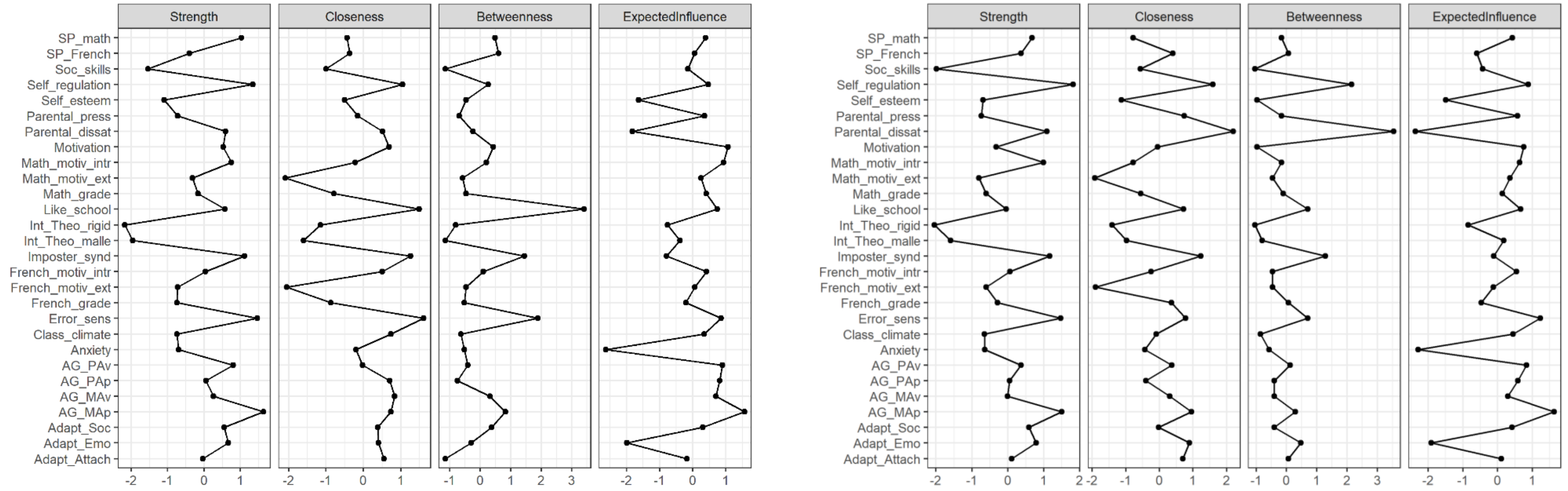

Note: centrality indices are standardised; Like_school – To like school or not; Anxiety – School anxiety; Self-regulation – Self-regulation; AG_MAp – Approach Master Goals; AG_MAv – Avoidance Master Goals; AG_PAp – Approach Performance Goals; AG_PAv – Avoidance Performance Goals; Emo_adapt – Emotional and personal adaptation; Soc_adapt – Social adapting; Attach_adapt – Attachment to the institution; Class_climate – Classroom climate; SP_French and SP_math– Self-perception in Maths and French; Soc_skills – Social skills; Self_esteem – Global self-esteem; Motivation – Motivation for cognitive activities; French_motiv_ext and Math_motiv_ext – External motivation for maths and French; French_motiv_intr and Math_motiv_intr – Intrinsic motivation for maths and french; Parental_dissat – Perception of parental dissatisfaction; Parental_press – Parental pressure; Error_sens – Error sensitivity; Imposter_synd – Feeling like an academic imposter; Int_Theo_rigid – Theory of entity intelligence (rigid); Int_Theo_malle – Theory of incremental intelligence (malleable); French_grade and Math_grade – Maths and French Competencies

Figure S8 Network plots with Rigid Theory of intelligence and external motivation in French and maths excluded

Figure S8 Network clusters for 4 groups with Rigid Theory of intelligence and external motivation in French and maths excluded

Panel A: M_MELEC

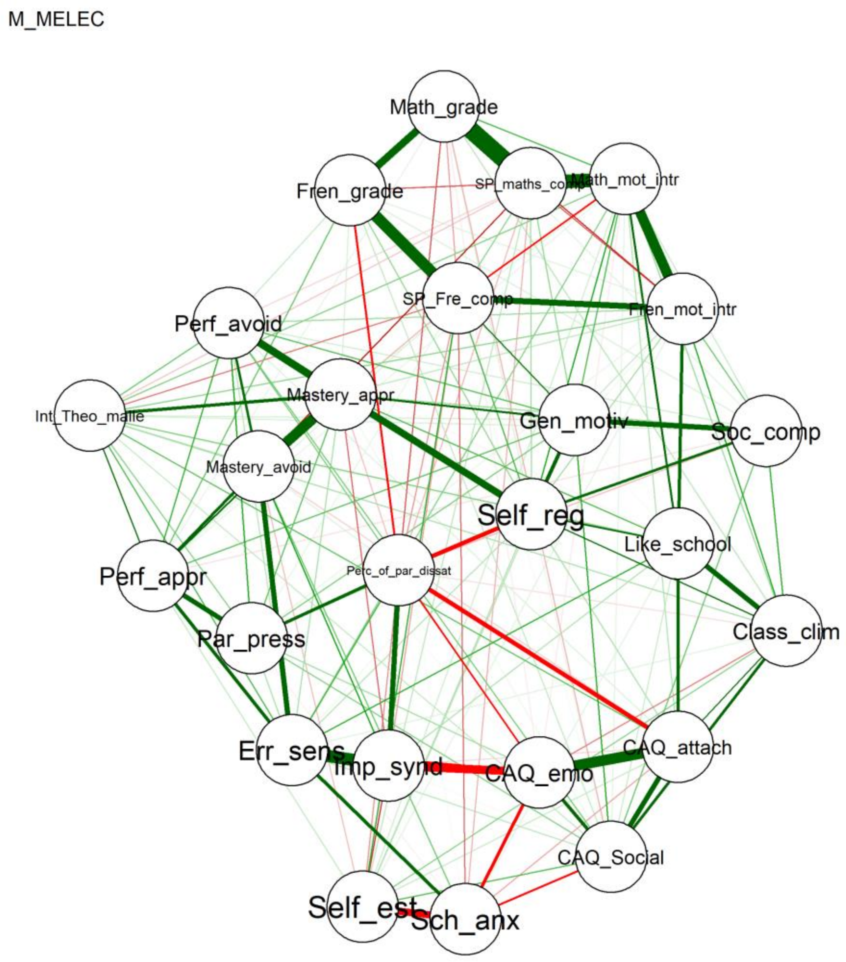

Panel B: F_ASSP

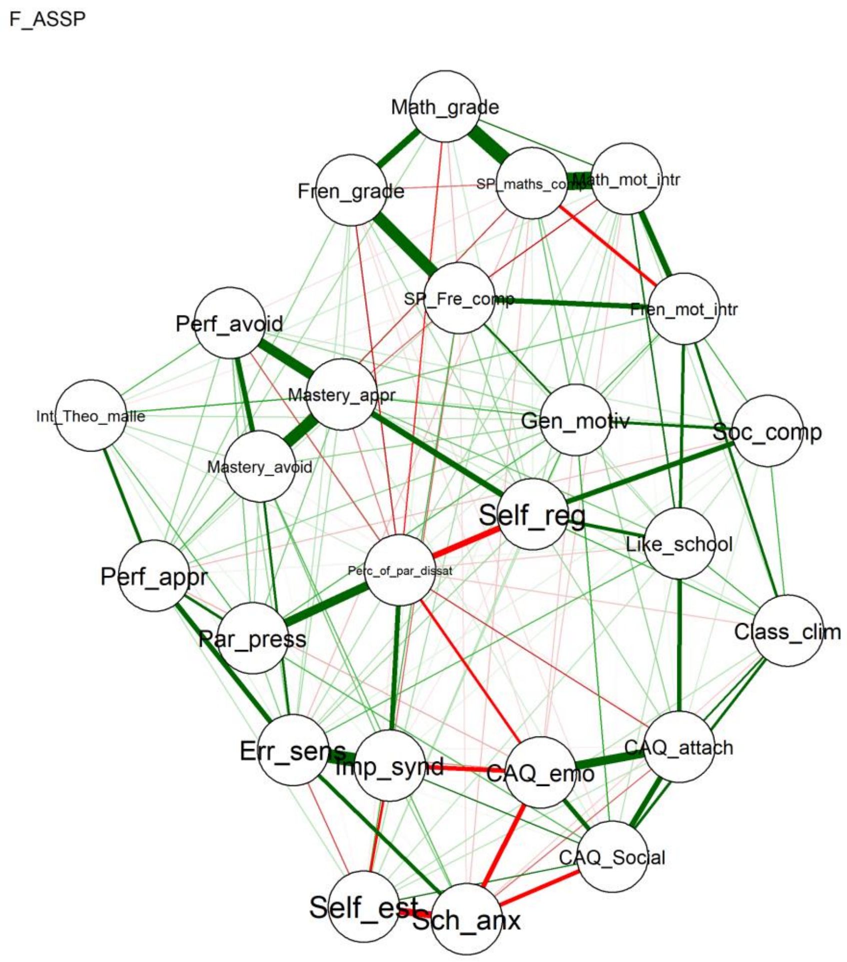

Panel C: M_Commerce

Panel D: F_Commerce

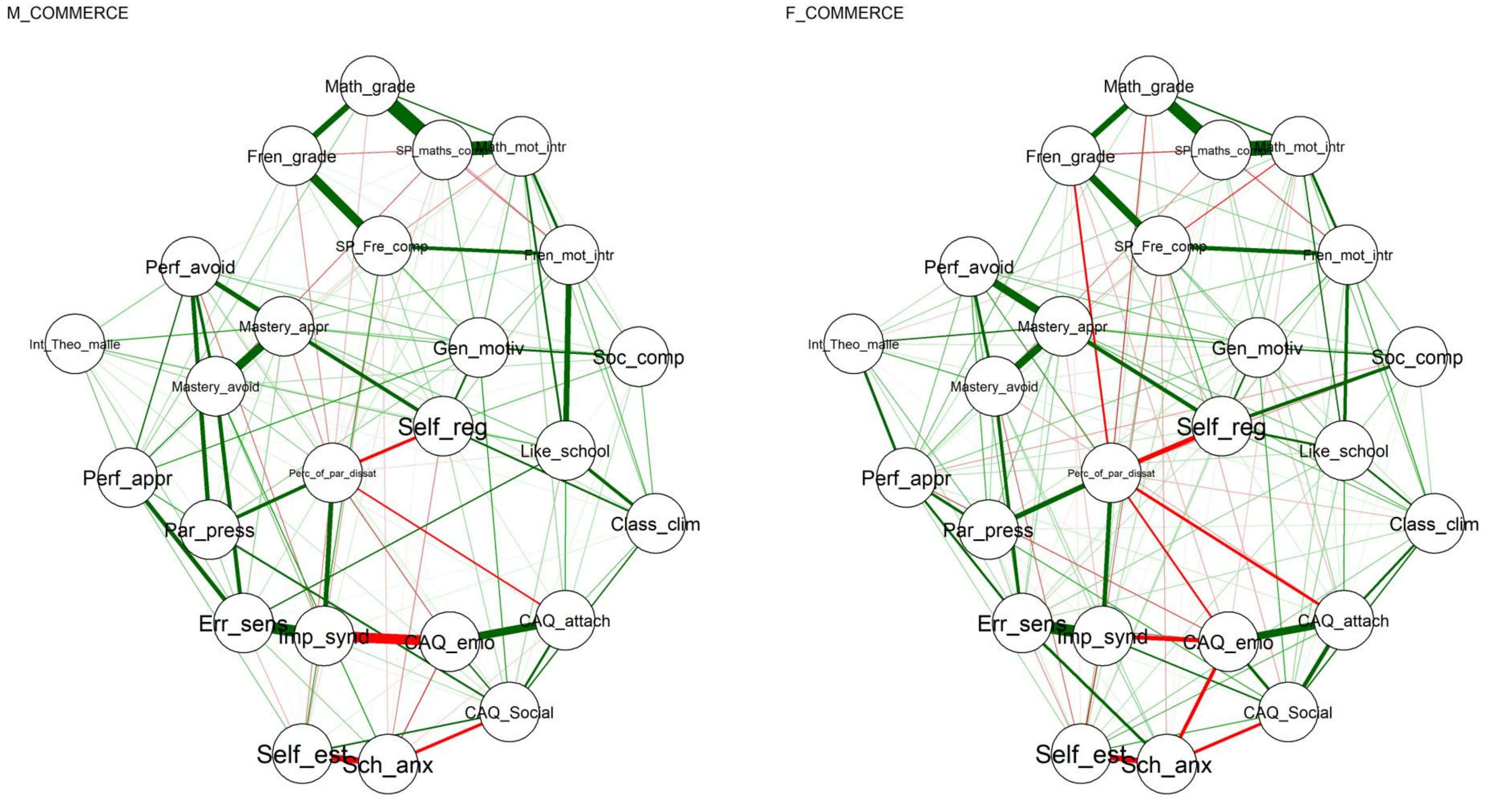

Note: Blue lines indicate positive connections; red lines indicate negative connections; the thickness of the lines represent the strength of the connection; clusters are color-coded, with different clusters marked with different colors. Like_school – To like school or not; Anxiety – School anxiety; Self-regulation – Self-regulation; AG_MAp – Approach Master Goals; AG_MAv – Avoidance Master Goals; AG_PAp – Approach Performance Goals; AG_PAv – Avoidance Performance Goals; Emo_adapt – Emotional and personal adaptation; Soc_adapt – Social adapting; Attach_adapt – Attachment to the institution; Class_climate – Classroom climate; SP_French and SP_math– Self-perception in Maths and French; Soc_skills – Social

skills; Self_esteem – Global self-esteem; Motivation – Motivation for cognitive activities; French_motiv_ext and Math_motiv_ext – External motivation for maths and French; French_motiv_intr and Math_motiv_intr – Intrinsic motivation for maths and french; Parental_dissat – Perception of parental dissatisfaction; Parental_press – Parental pressure; Error_sens – Error sensitivity; Imposter_synd – Feeling like an academic imposter; Int_Theo_rigid – Theory of entity intelligence (rigid); Int_Theo_malle – Theory of incremental intelligence (malleable); French_grade and Math_grade – Maths and French Competencies

Figure S9 Centrality plots with Rigid Theory of intelligence and external motivation in French and maths excluded

Figure S9 Centrality indices for network analysis in 4 groups with all variables except Rigid Theory of intelligence and external motivation in French and maths excluded

Panel A: M_MELEC

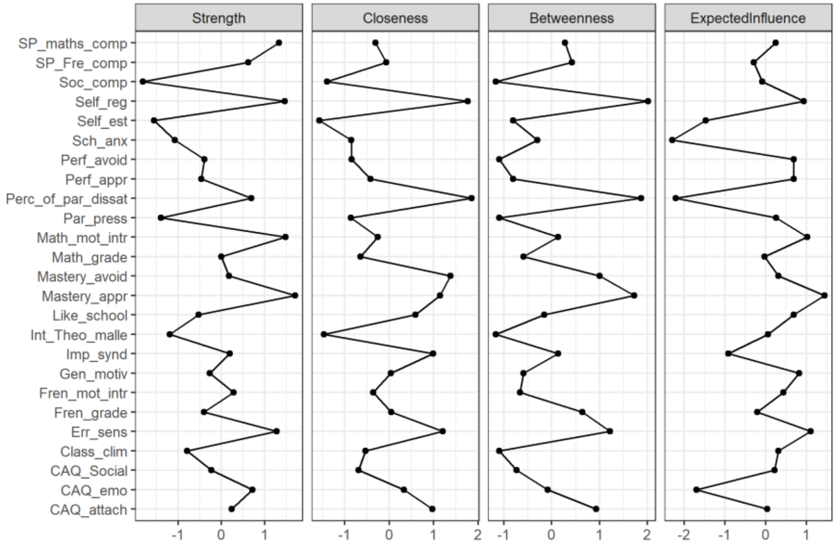

Panel B: F_ASSP

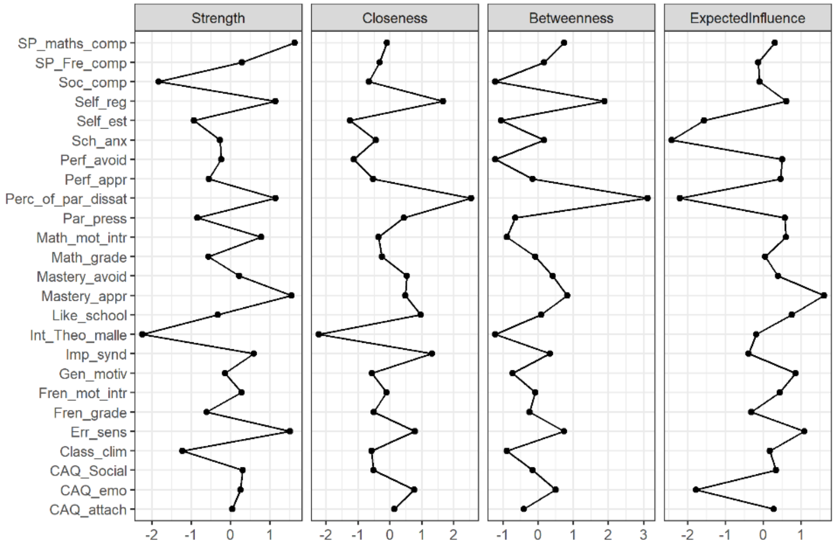

Panel C: M_Commerce

Panel D: F_Commerce

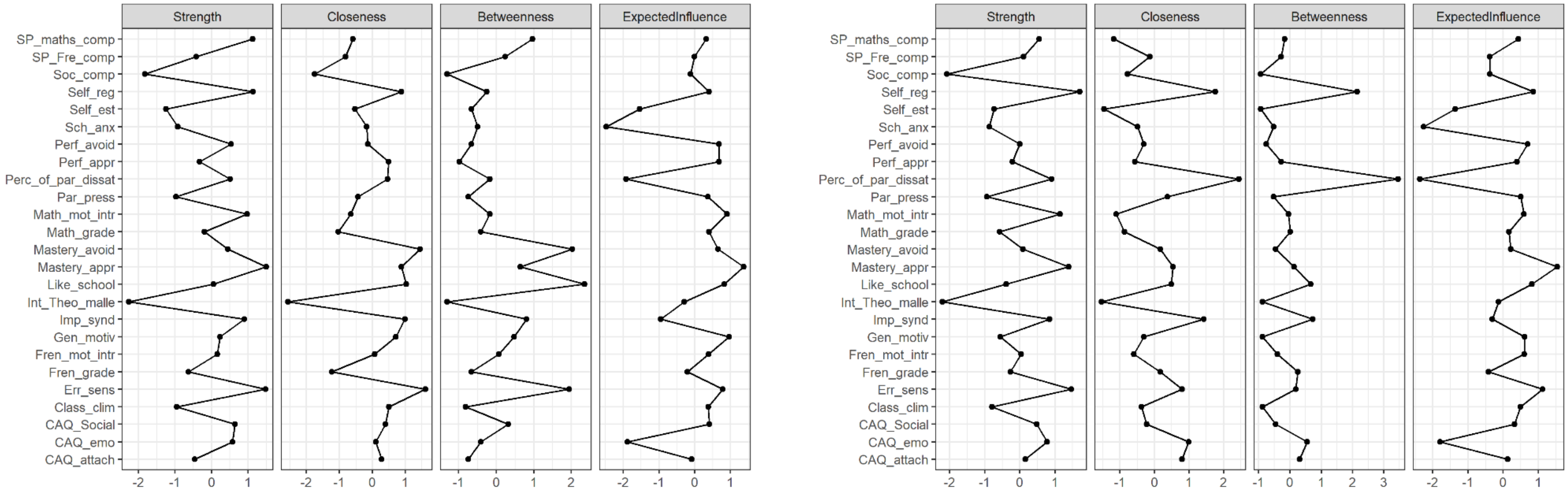

Note: centrality indices are standardised; Like_school – To like school or not; Anxiety – School anxiety; Self-regulation – Self-regulation; AG_MAp – Approach Master Goals; AG_MAv – Avoidance Master Goals; AG_PAp – Approach Performance Goals; AG_PAv – Avoidance Performance Goals; Emo_adapt – Emotional and personal adaptation; Soc_adapt – Social adapting; Attach_adapt – Attachment to the institution; Class_climate – Classroom climate; SP_French and SP_math– Self-perception in Maths and French; Soc_skills – Social skills; Self_esteem – Global self-esteem; Motivation – Motivation for cognitive activities; French_motiv_ext and Math_motiv_ext – External motivation for maths and French; French_motiv_intr and Math_motiv_intr – Intrinsic motivation for maths and french; Parental_dissat – Perception of parental dissatisfaction; Parental_press – Parental pressure; Error_sens – Error sensitivity; Imposter_synd – Feeling like an academic imposter; Int_Theo_rigid – Theory of entity intelligence (rigid); Int_Theo_malle – Theory of incremental intelligence (malleable); French_grade and Math_grade – Maths and French Competencies
